# Post-secondary Student Mental Health During COVID-19: A Meta-Analysis

**DOI:** 10.3389/fpsyt.2021.777251

**Published:** 2021-12-10

**Authors:** Jenney Zhu, Nicole Racine, Elisabeth Bailin Xie, Julianna Park, Julianna Watt, Rachel Eirich, Keith Dobson, Sheri Madigan

**Affiliations:** ^1^Department of Psychology, University of Calgary, Calgary, AB, Canada; ^2^Alberta Children's Hospital Research Institute, Calgary, AB, Canada

**Keywords:** depression, anxiety, mental health, post-secondary students, COVID-19

## Abstract

The COVID-19 pandemic has posed notable challenges to post-secondary students, causing concern for their psychological well-being. In the face of school closures, academic disruptions, and constraints on social gatherings, it is crucial to understand the extent to which mental health among post-secondary students has been impacted in order to inform support implementation for this population. The present meta-analysis examines the global prevalence of clinically significant depression and anxiety among post-secondary students during the COVID-19 pandemic. Several moderator analyses were also performed to examine sources of variability in depression and anxiety prevalence rates. A systematic search was conducted across six databases on May 3, 2021, yielding a total of 176 studies (1,732,456 participants) which met inclusion criteria. Random-effects meta-analyses of 126 studies assessing depression symptoms and 144 studies assessing anxiety symptoms were conducted. The pooled prevalence estimates of clinically elevated depressive and anxiety symptoms for post-secondary students during the COVID-19 pandemic was 30.6% (95% CI: 0.274, 0.340) and 28.2% (CI: 0.246, 0.321), respectively. The month of data collection and geographical region were determined to be significant moderators. However, student age, sex, type (i.e., healthcare student vs. non-healthcare student), and level of training (i.e., undergraduate, university or college generally; graduate, medical, post-doctorate, fellow, trainee), were not sources of variability in pooled rates of depression and anxiety symptoms during the pandemic. The current study indicates a call for continued access to mental health services to ensure post-secondary students receive adequate support during and after the COVID-19 pandemic.

**Systematic Review Registration:** PROSPERO website: https://www.crd.york.ac.uk/prospero/, identifier: CRD42021253547.

## Introduction

The coronavirus (COVID-19) pandemic has disrupted the lives of individuals around the world. Physical-distancing measures and quarantine orders implemented were intended to prepare for, and mitigate the risk of, an overburdened healthcare system. However, an unintended consequence of these protective measures is an increased risk for mental illness. Indeed, one of the largest and most sustained effects of the COVID-19 pandemic is estimated to be its negative effects on the mental health and well-being of citizens (1–4). Several emerging meta-analyses of general population samples show that rates of mental illness have increased during the COVID-19 pandemic (1, 5). Further, large population-based samples with longitudinal pre-pandemic data have shown that the mental health of certain subgroups of the population have deteriorated more rapidly, including individuals aged 18–24 (3), many of whom are post-secondary students.

Post-secondary students may be uniquely at increased risk for mental illness during the pandemic due to university/college closures, academic disruptions, and social restrictions. Extensive research has been conducted on the mental health of post-secondary students during the COVID-19 pandemic, and prevalence rates have varied widely, from 1.3–100% for clinically elevated depression and 1.1–100% for clinically elevated anxiety (6, 7). Ascertaining more precise estimates of clinically significant depression and anxiety symptoms among post-secondary students globally during the COVID-19 pandemic will be important for informing how supports can be allocated to young adults. To this end, we conducted a systematic review and meta-analysis of research amassed to date. We also conducted demographic and methodological study quality moderator analyses in order to identify under what circumstances and for whom prevalence rates of depression and anxiety may be higher or lower. These moderator analyses may inform practice and health policy initiatives more reliably and be used to guide future research.

### Depression and Anxiety Symptoms in Post-secondary Students

Depression and anxiety are two of the most common mental illnesses in the general population and represent leading causes of disease burden worldwide (8). Depression is characterized by overwhelming feelings of sadness, hopelessness, as well as lack of interest, pleasure, and/or motivation. Depression often has associated physical symptoms, such as sleep, appetite, and concentration difficulties. Anxiety includes symptoms such as excessive worry, physiological hyperarousal, and/or debilitating fear. Existing meta-analyses have demonstrated that, prior to COVID-19, 23.8% of Chinese university students and 24.4% of university students living in low- and middle-income countries experienced symptoms of depression (9, 10). Further, 33.8% of university students globally experienced at least mild symptoms of anxiety (11) and a meta-analysis of Iranian university students found 33% of students experienced mild to severe anxiety (12). A study of over 43,000 Canadian college students found 14.7 and 18.4% of students were diagnosed or treated for depression and anxiety, respectively, in the past 12 months (13).

There are several reasons to expect that depression and anxiety will rise due to the COVID-19 pandemic. Being quarantined is associated with negative psychological symptoms, such as stress, loneliness, confusion, and anger (14, 15). Fear of contamination, or fear of death to self or loved ones, can lead to efforts to increase self-isolation (16). The unpredictable and uncontrollable nature of COVID-19 can also increase mental distress. When social capital, such as social support, community integration, social norms, as well as family rituals, norms, and values are limited or inhibited, disruptions to emotional and behavioral regulation are likely to occur (16–18). Unique to post-secondary students, stressors include a fear of class cancellation and missed milestones (e.g., graduation), which could lead to increased psychological distress (19). Moreover, peer relationships represent a crucial and prominent source of social support among emerging adults (20). Given academic closures and isolation measures, students were distanced from a crucial support network during the COVID-19 pandemic.

To date, several meta-analyses have attempted to synthesize pooled prevalence estimates of depression and anxiety among post-secondary students during the COVID-19 pandemic. Research examining depression symptoms have found pooled prevalence rates that range from 26 to 34% (21–24) and anxiety symptoms that range from 28 to 31% (21, 24, 25). However, there are several limitations of the previous meta-analyses. First, their inclusion criteria often did not specify the need for moderate-to-severe symptoms, which are considered to indicate “clinically elevated” mental distress. Second, several of the meta-analyses examined specific student populations (e.g., nursing or medical students) who may experience higher rates of mental illness during the COVID-19 pandemic due to stress from frontline clinical work (26) and may, in turn, inflate prevalence estimates. Third, several of the existing meta-analyses did not explore sources of between-study variability (i.e., moderators) in prevalence estimates. A central goal of a meta-analysis is to conduct moderator analyses to determine if between-study variability can be attributed to methodological or demographic factors. Finally, existing meta-analyses have only synthesized data from a portion of time over the course of the pandemic. The current meta-analysis addresses the above-mentioned issues by synthesizing data on clinically elevated symptoms of depression and anxiety (i.e., moderate to severe) which is more consistent with large-scale research reporting on the prevalence of mood and anxiety disorders [e.g., (27)] and studies evaluating the global burden of diseases, which are typically based on the proportion of individuals who meet the threshold for DSM/ICD criteria (28). The present meta-analysis also addresses gaps in existing literature by conducting moderator analyses and includes studies on all populations of post-secondary students well over a year into the COVID-19 pandemic.

### Potential Moderators of Prevalence Rates

Within the context of a meta-analysis, moderator analyses can ascertain whether certain populations of post-secondary students are at higher risk for mental health symptoms during the COVID-19 pandemic, as well as whether certain study-level characteristics, such as methodological characteristics, explain variability in prevalence estimates. As mentioned, compared to studies investigating post-secondary students broadly, the mental health of students enrolled in healthcare fields involved in clinical work may have been disproportionately affected by COVID-19 due to engaging in frontline clinical training in addition to the pandemic-related changes affecting all students, such as academic closures and online learning. Further, mental illness rates have been found to differ based on level of training. A previous meta-analysis found higher rates of mental illness among undergraduate students relative to graduate students during the COVID-19 pandemic (29). Differing rates of mental illness across levels of training could be the result of the distinct stressors at each level, which could be exacerbated by the pandemic. For example, undergraduate students are often adjusting to increased independence during an age that coincides with the onset of many mental illnesses (30). Graduate students, however, may be focused on academic work and have longer work hours which may limit the amount of time dedicated to protective factors such as social activities and hobbies (31). Another source of between-study variability could include methodological factors. For example, it is likely that the desire for rapid information about mental health during COVID-19 has led to less rigorous methodologies [e.g., convenience sampling; (32)], which may explain between-study heterogeneity. Geographical region may also increase or decrease the prevalence of mental illness during the pandemic. A meta-analysis of child and adolescent mental illness during the COVID-19 pandemic found higher rates of anxiety symptoms in European countries compared to East Asian countries (4). Rates may vary across geographical region as certain countries or regions have more accepting attitudes toward mental illness (33). In addition, countries have varied in terms of COVID-19 infection rates, strictness of quarantine and self-isolation orders, and governmental responses to the pandemic, all of which could impact reports of mental distress. Rates may also vary over the course of the pandemic, such that continued social isolation and school disruptions may have more negative effects on mental health over time. Indeed, existing research has found that rates of mental illness were higher later in the pandemic compared to the beginning of the pandemic (4, 34). More generally, it is also well-established that symptoms of depression and anxiety are more common among females than males (33) and the age of onset for both depression and anxiety disorders begins in young adulthood (35), thus sex and age will also be examined as moderators.

### The Current Study

The aim of the current meta-analysis was to provide estimates of the global prevalence of clinically elevated depression and anxiety symptoms during the COVID-19 pandemic among post-secondary student samples. It was hypothesized that depression and anxiety have increased on account of the COVID-19 pandemic, compared to prior global estimates. Methodological study quality, type of student (i.e., healthcare vs. non-healthcare), level of training (i.e., undergraduate, university or college generally; graduate, medical, post-doctorate, fellow, trainee), as well as participant sex, age, month data collection was completed, and geographical region were explored as potential moderating factors that may amplify or attenuate prevalence estimates.

## Methods

### Search Strategy and Selection Criteria

This review is reported in accordance with the Preferred Reporting Items for Systematic Reviews and Meta-Analyses (PRISMA) 2020 guidelines (36) and the PRISMA-S extension (37). The protocol for this review was developed by the authors and registered with the PROSPERO International Prospective Register of Systematic Reviews (CRD42021253547). Searches were conducted in MEDLINE (Ovid), EMBASE (Ovid), APA PsycINFO (Ovid), Cochrane Central Register of Controlled Trials (Ovid), ERIC (EBSCOhost), and Education Research Complete (EBSCOhost) by a health sciences librarian on May 3, 2021. Search strategies combined search terms falling under three themes: (1) mental health and illness (including, anxiety and depression); (2) COVID-19; and (3) students (see Supplementary Tables 1–5 for full search strategies in each database). The search included students broadly with the understanding that results could be more deliberately limited to the post-secondary audience during the screening phase. Terms were searched both as keywords and as database subject headings as appropriate. Both adjacency operators and truncation were used to capture phrasing variations in keyword searching. No language or date restrictions were applied. References of relevant studies were reviewed manually for additional pertinent articles. Using Covidence software, three authors reviewed all titles, abstracts, and full text articles emerging from the search strategy to determine eligibility for inclusion. All abstracts were reviewed by at least two independent coders. Disagreements were resolved to consensus *via* expert review by the first author. All studies identified in the abstract review as meeting inclusion criteria, underwent full text review by five coders to ensure that all inclusion criteria were met. Thirty percent of full texts were reviewed by two independent coders and random agreement probabilities ranged from 0.72 to 0.90.

### Data Extraction

Studies meeting inclusion criteria during full text review underwent data extraction. In this phase, prevalence data on clinically elevated anxiety and depression symptoms were recorded. We also extracted data on the following moderators: (1) study quality (see below); (2) participant age (continuously as a mean); (3) sex (% male in a sample); (4) type of student (healthcare; non-healthcare); (5) level of training (undergraduate, university or college generally; graduate, medical, post-doctorate, fellow, trainee), (6) time of data collection (i.e., month in 2020) and (7) geographical region (e.g., East Asia, Europe, North America). Twenty percent of included studies underwent data extraction by a second coder to verify judgements for correctness and accuracy (random agreement probabilities ranged from 0.84 to 1.00). Discrepancies were resolved *via* discussion and attainment of consensus coding.

### Study Quality

A 5-item study quality measure was used, based on modified versions of the National Institute of Health Quality Assessment Tool for Observation Cohort and Cross-Sectional Studies and the Newcastle-Ottawa Quality Assessment Scale (38) for cross-sectional studies (scores ranged from 0 to 5). The following criteria were applied: (1) outcome was assessed with a validated measure of depression and/or anxiety; (2) study was peer-reviewed vs. unpublished; (3) study had a response rate of at least 50%; (4) depression or anxiety was assessed objectively (i.e., diagnostic interview); (5) the study had sufficient exposure time to COVID-19 (i.e., at least 1 week since the onset of COVID-19 in the specific country where the study was conducted). Studies were given a score of 0 (no) or 1 (yes) for each criterion and a summed score out of 5. When information was not provided by the study authors, it was marked as 0 (no). The coding protocol for the quality scoring can be found in Supplementary Table 6.

### Data Analysis

Extracted data were entered into Comprehensive Meta-Analysis [CMA version 3.0; (39)]. Pooled prevalence rates were computed with associated 95% confidence intervals (CIs) around the estimate. CMA transforms the prevalence into a logit event rate (i.e., represented as 0.XX but interpreted as prevalence = XX%) with a computed standard error. Subsequently, event rates are weighted by the inverse of their variance, giving greater weight to studies with larger sample sizes. Finally, logits are retransformed into proportions to facilitate ease of interpretation.

Random-effects models, which assume that variations observed across studies exist because of differences in samples and study designs, were used. To assess for between-study heterogeneity, the *Q* and *I*^2^ statistics were computed. A significant *Q* statistic suggests that study variability is greater than sampling error and that moderator analyses should be explored (40). The *I*^2^ statistic, which ranges from 0 to 100%, examines the rate of variability across studies (41). Typically, when *I*^2^ values are > 75%, moderator analyses should be explored (41). As recommended by Borenstein et al. (39), categorical moderators were conducted when *k* ≥ 10 with a cell size of *k* > 3 for each categorical comparison. Random-effect meta-regression analyses were conducted with restricted maximum likelihood estimation for all continuous moderators. Egger's test and visual examination of funnel plots was utilized to identify publication bias (42). The set threshold for significance of moderators was *p* < 0.05.

## Results

**Figure 1:**
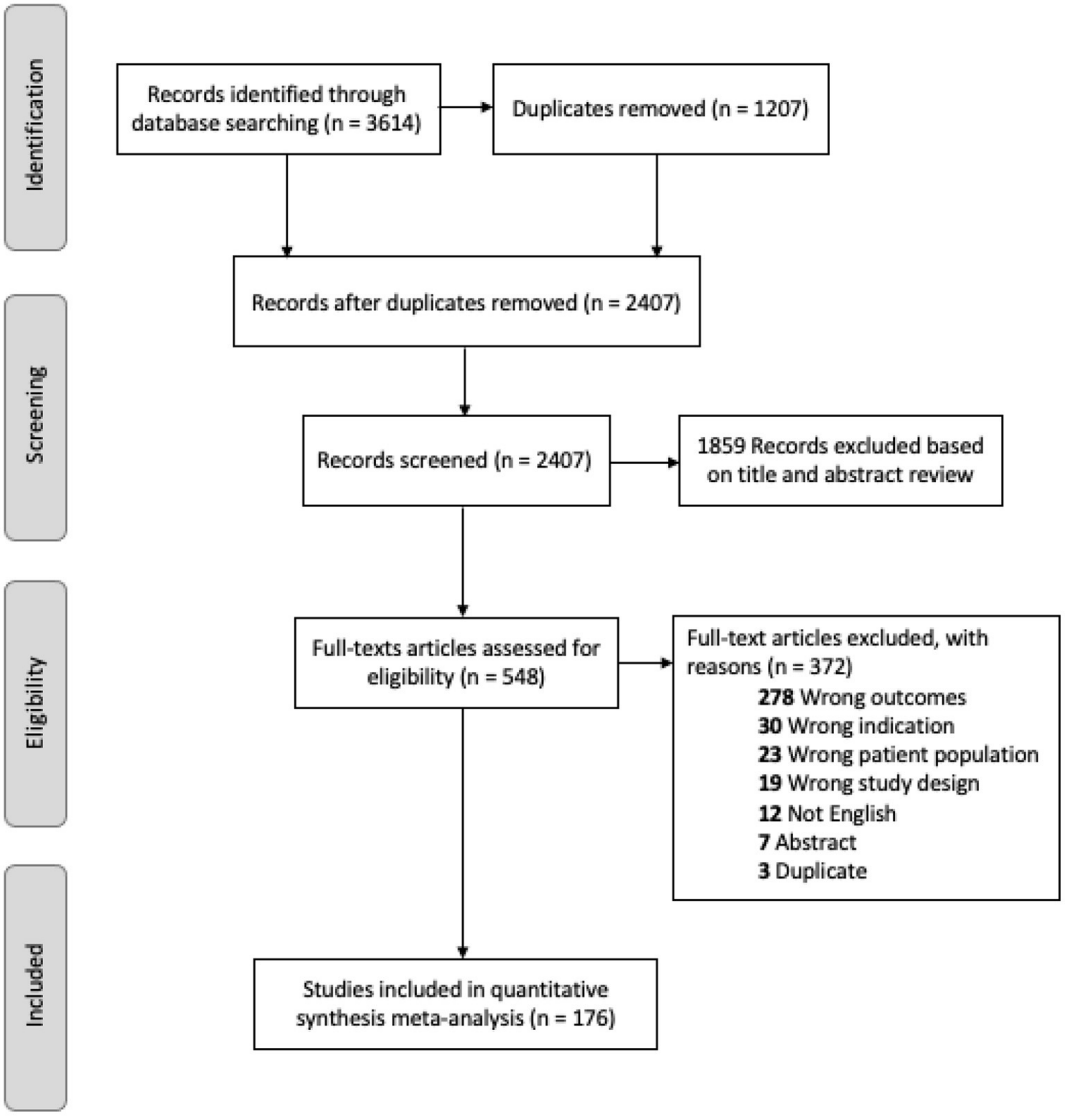
PRISMA diagram of review search strategy.

As illustrated in the PRISMA flow diagram (see Figure 1), the electronic search yielded 3,614 records. After removing 1,207 duplicates, 548 full-text articles were retrieved for evaluation against inclusion criteria and 176 non-overlapping studies met full inclusion criteria.

### Study Characteristics

The present meta-analysis included 176 studies, 126 of which reported clinically significant depression symptoms and 144 reported on clinically significant anxiety symptoms. As detailed in Table 1, across all 176 studies, 1,732,456 participants were included, with 35.6% being male and a mean age of 21.8 years (age range, 18.5–31.5). Forty-eight studies (27.3%) were from East Asia, 40 (22.7%) from Europe, 35 (19.9%) from South Asia, 18 (10.2%) from Middle East, 17 (9.7%) from North America, eight (4.5%) from Southeast Asia, four (2.3%) from Africa, three (1.7%) from Central America, one (0.6%) from Oceania, and two were from multiple geographical regions. The mean study quality score was 3.5 out of 5 (range: 2–4; see Supplementary Table 7). Specifically, 176 (100%) studies used validated measures; 176 (100%) were peer-reviewed, 102 (58.0%) had a response rate ≥ 50%, no studies (0%) used diagnostic interviews to assess clinically elevated anxiety or depression, and 165 (93.8%) of studies had sufficient exposure time to COVID-19.

**Table 1:** Characteristics of studies included.

**References**	***N* ^a^**	**Mean age (years)**	**% male**	**Country**	**Mental health measured**	**Name of mental health measures**	**Month of data collection^b^**	**Published**	**Health-care student**	**Level of training^c^**	**Study design**
Abas et al. (43)	478	21.55	72.00	Sudan	Anx	BAI	May	Yes		Undergrad	Cross.
Ahmed et al. (44)	1,445	-	29.40	Pakistan	Anx, Dep	GAD-7, PHQ-9	May—July	Yes	Yes	Postgrad	Cross.
Akinkugbe et al. (45)	426	-	37.70	USA	Anx	GAD-7	April—May	Yes	Yes	Postgrad	Cross.
Alkhamees and Aljohani (46)	336	-	-	Saudi Arabia	Anx, Dep	DASS-21	April	Yes	-	Undergrad	Cross.
Alqudah et al. (47)	736	20.97	24.90	Jordan	Anx	HAM-A	April—May	Yes	-	Undergrad	Cross.
Alsairafi et al. (48)	298	-	10.40	Kuwait	Anx, Dep	GAD-7, PHQ-9	May—July	Yes	-	Undergrad	Cross.
Amatori et al. (49)	159	23.00	50.94	Italy	Dep	PHQ-9	April	Yes	-	Undergrad	Cross.
Amendola et al. (50)	676	25.00	24.00	Switzerland	Anx	GAD-7	April	Yes	-	Undergrad	Long.
Amerio et al. (51)	8,177	22.02	50.10	Italy	Dep	PHQ-9	April—May	Yes	-	Undergrad	Cross.
Aslan et al. (52)	358	23.00	42.46	Turkey	Anx, Dep	GAD-7, PHQ-8	May—June	Yes	-	Undergrad	Cross.
Aylie et al. (53)	314	-	63.40	Ethiopia	Anx, Dep	DASS-21	May—June	Yes	-	Undergrad	Cross.
Balhara et al. (54)	128	19.60	40.00	India	Anx, Dep	GAD-7, PHQ-9	-	Yes	-	Undergrad	Cross.
Baloch et al. (55)	494	-	39.00	Pakistan	Anx	SAS	May—June	Yes	-	Undergrad	Cross.
Bashir et al. (56)	523	24.61	20.10	Pakistan	Anx, Dep	GAD-7, PHQ-9	August—September	Yes	-	Undergrad	Cross.
Batais et al. (57)	322	21.92	46.90	Saudi Arabia	Anx	GAD-7	March	Yes	Yes	Postgrad	Cross.
Biber et al. (58)	1,640	-	38.60	USA	Anx	GAD-7	April	Yes	-	Undergrad	Cross.
Bilgi et al. (59)	178	21.00	28.65	Turkey	Anx, Dep	GAD-7, PHQ-9	June	Yes	Yes	Postgrad	Cross.
Biswas and Biswas (60)	209	20.33	12.44	India	Anx	GAD-7	-	Yes	-	Undergrad	Cross.
Blake et al. (61)	99	20.36	13.10	UK	Anx	GAD-7	July—October	Yes	-	Undergrad	Cross.
Bolatov et al. (62)	798	20.31	24.30	Kazakhstan	Anx, Dep	GAD-7, PHQ-9	April	Yes	Yes	Postgrad	Cross.
Bourion-Bedes et al. (63)	3,936	21.70	29.40	France	Anx	GAD-7	May	Yes	-	Undergrad	Cross.
Brett et al. (64)	151	-	24.80	Australia	Anx	GAD-7	March—May	Yes	-	Undergrad	Cross.
Cam et al. (65)	1,095	21.72	25.50	Turkey	Anx, Dep	DASS-21	May 2020	Yes	-	Undergrad	Cross.
Campos et al. (66)	66	21.70	24.2	Brazil	Anx, Dep	DASS-12	May—June	Yes	Yes	Undergrad	Cross.
Chakraborty et al. (67)	168	24.00	19.00	India	Dep	PHQ-9	May 2020	Yes	Yes	Postgrad	Cross.
Chen et al. (68)	361,969	-	40.30	China	Dep	PHQ-9	February	Yes	-	Undergrad	Cross.
Chi et al. (69)	2,038	20.56	37.00	China	Anx, Dep	SAS, PHQ-9	February	Yes	-	Undergrad	Cross.
Cici and Yilmazel (70)	322	20.80	23.60	Turkey	Anx	BAI	March—April	Yes	Yes	-	Cross.
Cuschieri and Calleja Agius (71)	172	-	33.70	Malta	Anx	GAD-7	April—May	Yes	Yes	Postgrad	Cross.
Dangal and Bajracharya (72)	96	20.95	20.9	Nepal	Anx	GAD-7	-	Yes	-	-	Cross.
Das et al. (73)	208	-	56.70	Bangladesh	Anx, Dep	GAD-7, PHQ-9	April—May	Yes	-	-	Cross.
Deng et al. (6)	1,607	-	64.80	China	Anx, Dep	DASS-21	May 2020	Yes	-	Undergrad	Cross.
Dhar et al. (74)	15 543	-	66.70	Bangladesh	Anx	GAD-7	-	Yes	-	Undergrad	Cross.
Diaz-Jimenez et al. (75)	365	23.22	9.90	Spain	Anx	DASS-21	May 2020	Yes	-	Undergrad	Cross.
Dratva et al. (76)	2,223	26.4	30.00	Switzerland	Anx	GAD-7	April 2020	Yes	-	Undergrad	Cross.
Du et al. (77)	2,254	22.50	30.80	China, Ireland, Malaysia, South Korea, Taiwan, Netherlands, USA	Anx	GAD-7	April—May	Yes	-	-	Cross.
Dun et al. (78)	12,889	20.00	20.00	China	Dep	BDI-II	May	Yes	-	Undergrad	Cross.
Elhadi et al. (79)	2,430	23.30	21.00	Libya	Anx, Dep	GAD-7, PHQ-9	April—May	Yes	Yes	Postgrad	Cross.
El-Monshed et al. (80)	612	20.00	38.20	Egypt	Anx, Dep	DASS-21	May – June	Yes	-	Undergrad	Cross.
Essadek and Rabeyron (81)	8,004	21.70	32.60	France	Anx, Dep	GAD-7, PHQ-9	April	Yes	-	Undergrad	Cross.
Evans et al. (82)	254	19.76	12.60	UK	Dep	HADS	April—May	Yes	-	Undergrad	Long.
Faisal et al. (83)	874	22.83	63.80	Bangladesh	Anx, Dep	GAD-7, CES-D	April	Yes	-	Undergrad	Cross.
Far Abid Hossain et al. (84)	474	-	61.80	Bangladesh	Anx	SAS	May—June	Yes	-	-	Cross.
Fawaz and Samaha (85)	520	21.03	38.70	Lebanon	Anx, Dep	DASS-21	April	Yes	-	Undergrad	Cross.
Feng et al. (86)	1,346	19.76	27.00	China	Anx, Dep	GAD-7, PHQ-9	Febuary	Yes	-	Undergrad	Cross.
Feng et al. (87)	219	23.17	25.10	China	Anx	GAD-7	March—April	Yes	-	-	Cross.
Fruehwirth et al. (88)	419	18.90	-	USA	Anx, Dep	GAD-7, PHQ-8	June—July	Yes	-	Undergrad	Long.
Fu et al. (89)	89 588	-	43.75	China	Anx	GAD-7	May—June	Yes	-	Undergrad	Cross.
Garvey et al. (90)	198	-	32.80	Spain	Anx	GAD-7	April	Yes	-	Undergrad	Cross.
Gas et al. (91)	699	21.31	35.30	Turkey	Anx, Dep	DASS-21	May—July	Yes	Yes	Postgrad	Cross.
Ge et al. (92)	2,009	-	49.03	China	Anx	GAD-7	Febuary	Yes	-	Undergrad	Long.
Gecaite-Stonciene et al. (93)	619	22.00	7.10	Lithuania	Anx, Dep	GAD-7, PH-9	May—November	Yes	-	Undergrad	Cross.
Generali et al. (94)	399	23.45	43.10	Italy	Anx	GAD-7	April—May	Yes	Yes	Postgrad	Cross.
Ghazawy et al. (95)	1,335	-	38.20	Egypt	Anx, Dep	DASS-21	June 2020	Yes	-	Undergrad	Cross.
Giusti et al. (96)	103	22.50	18.40	Italy	Anx, Dep	SAS, BDI-II	March—May	Yes	-	Undergrad	Cross.
Graupensperger et al. (97)	135	19.84	37.00	USA	Dep	PROMIS	February—April	Yes	-	Undergrad	Long.
Guo et al. (98)	852	-	-	USA	Anx	GAD-7	June—August	Yes	Yes	Postgrad	Cross.
Hakami et al. (99)	697	21.76	45.30	Saudi Arabia	Anx, Dep	DASS-21	April	Yes	Yes	Postgrad	Cross.
Halperin et al. (100)	1,428	22.30	32.40	USA	Anx, Dep	GAD-7, PHQ-9	April	Yes	Yes	Postgrad	Cross.
Hamza et al. (101)	733	18.52	25.00	Canada	Anx, Dep	GAD-7, CES-D	April	Yes	-	Undergrad	Long.
Imran et al. (102)	10,178	31.50	43.30	Pakistan	Anx, Dep	GAD-7, PHQ-9	April—May	Yes	Yes	Postgrad	Cross.
Islam et al. (103)	3,122	21.40	59.50	Bangladesh	Anx, Dep	DASS-21	April	Yes	-	Undergrad	Cross.
Islam et al. (104)	476	-	67.20	Bangladesh	Anx, Dep	GAD-7, PHQ-9	May	Yes	-	Undergrad	Cross.
Jia et al. (105)	740	-	38.11	China	Anx	SAS	February	Yes	-	Undergrad	Cross.
Jin et al. (106)	847	20.09	22.40	China	Anx, Dep	DASS-21	March	Yes	-	Undergrad	Cross.
Jindal et al. (107)	664	-	47.60	India	Anx	GAD-7	May	Yes	Yes	Undergrad	Cross.
Jones et al. (108)	2,282	-	42.10	USA	Anx, Dep	PHQ-4	April	Yes	Yes	Undergrad	Cross.
Joshi et al. (109)	2,088	-	23.00	India	Anx	GAD-7	-	Yes	-	-	Cross.
Juchnowicz et al. (110)	2,172	22.10	27.01	Poland	Anx, Dep	DASS-21	April	Yes	-	Undergrad	Cross.
Kadam et al. (111)	60	-	12.00	India	Anx	HAM-A	-	Yes	-	Undergrad	Cross.
Kalkan Ugurlu et al. (112)	411	20.60	20.70	Turkey	Anx, Dep	DASS-42	July	Yes	Yes	-	Cross.
Kalok et al. (113)	772	-	28.40	Malaysia	Anx, Dep	DASS-21	April	Yes	Yes	Postgrad	Cross.
Kamaludin et al. (114)	983	-	33.60	Malaysia	Anx	SAS	April—May	Yes	-	Undergrad	Cross.
Kannampallil et al. (115)	393	-	45.00	USA	Anx, Dep	DASS-21	April	Yes	Yes	Postgrad	Cross.
Kaparounaki et al. (116)	1,000	22.07	30.99	Greece	Dep	CES-D	April	Yes	-	Undergrad	Cross.
Kassir et al. (117)	73	-	27.40	Lebanon	Anx, Dep	GHQ-28	June—September	Yes	-	Undergrad	Cross.
Khoshaim et al. (118)	400	-	24.80	Saudi Arabia	Anx	SAS	April—June	Yes	-	Undergrad	Cross.
Kibbey et al. (119)	641	20.10	27.30	USA	Anx, Dep	DASS-21	April—May	Yes	-	Undergrad	Cross.
Kohls et al. (120)	3,382	23.98	28.6	Germany	Dep	PHQ-9	July—August	Yes	-	Undergrad	Cross.
Kuman Tuncel et al. (121)	3,105	22.37	43.30	Turkey	Anx	BAI	April—May	Yes	Yes	Postgrad	Cross.
Lai et al. (122)	124	-	36.30	UK, USA	Anx, Dep	PHQ-4, PH-4	April—May	Yes	-	Undergrad	Cross.
Lan et al. (123)	304	-	71.40	Vietnam	Anx, Dep	DASS-18	March	Yes	-	Undergrad	Cross.
Le Vigouroux et al. (124)	1,297	21.27	20.66	France	Anx, Dep	HADS	March—May	Yes	-	Undergrad	Cross.
Lee et al. (125)	1,410	-	26.00	USA	Anx, Dep	GAD-7, PROMIS-D	March—May	Yes	-	-	Cross.
Li et al. (126)	68,685	-	36.80	China	Anx, Dep	GAD-7, PHQ-9	February	Yes	-	Undergrad	Long.
Li et al. (127)	7,747	20.74	50.95	China	Anx, Dep	GAD-7, PHQ-9	February—March	Yes	-	Undergrad	Cross.
Li et al. (128)	1,168	-	65.07	China	Anx	GAD-7	April—June	Yes	-	Undergrad	Cross.
Li et al. (129)	6,348	-	9.63	China	Anx, Dep	GAD-7, PHQ-9	March	Yes	Yes	Undergrad	Cross.
Liang et al. (130)	4,164	-	52.00	China	Dep	PHQ-9	February	Yes	-	Undergrad	Cross.
Lin et al. (131)	628	20.17	35.20	China	Dep	CES-D	March	Yes	-	Undergrad	Cross.
Lin et al. (132)	2,086	-	-	China	Anx	STAI-6	April	Yes	Yes	Postgrad	Cross.
Lischer et al. (133)	557	27.00	36.20	Switzerland	Anx	PHQ-4	April—May	Yes	-	Undergrad	Cross.
Liu et al. (134)	217	21.70	41.50	China	Anx, Dep	GAD-7, PHQ-9	February—April	Yes	Yes	Postgrad	Cross.
Lopez-Castro et al. (135)	909	-	30.80	USA	Anx, Dep	GAD-7, PHQ-9	May	Yes	-	Undergrad	Cross.
Ma et al. (136)	746,217	-	44.40	China	Anx, Dep	GAD-7, PHQ-9	February	Yes	-	Undergrad	Cross.
Majumdar et al. (137)	325	22.10	39.07	India	Dep	CES-D	April—May	Yes	-	-	Cross.
Manjareeka and Pathak (138)	101	19.70	63.37	India	Anx	STAI-S	February—May	Yes	Yes	Postgrad	Long.
Mechili et al. (139)	892	-	11.4	Albania	Dep	PHQ-9	March—April	Yes	-	Undergrad	Cross.
Medeiros et al. (140)	113	21.46	23.00	Brazil	Anx, Dep	HADS	May	Yes	Yes	Postgrad	Cross.
Mekonen et al. (141)	338	24.70	56.20	Ethiopia	Anx, Dep	DASS-21	November	Yes	-	Undergrad	Cross.
Meng et al. (142)	3,304	21.18	39.39	China	Anx, Dep	GAD-7, PHQ-9	February	Yes	-	Undergrad	Cross.
Miskulin et al. (143)	347	-	-	Brazil	Dep	HADS	March—June	Yes	Yes	Postgrad	Cross.
Moayed et al. (144)	207	-	69.08	Iran	Anx, Dep	DASS-21	February—March	Yes	Yes	Postgrad	Cross.
Mridul et al. (145)	159	-	-	India	Anx, Dep	DASS-21	July	Yes	-	Undergrad	Cross.
Mushquash and Grassia (146)	131	20.32	19.08	Canada	Dep	PHQ-9	May	Yes	-	Undergrad	Cross.
Nakhostin-Ansari et al. (147)	323	23.73	47.70	Iran	Anx, Dep	BAI, BDI	April	Yes	Yes	Postgrad	Cross.
Naser et al. (148)	1,165	-	46.20	Jordan	Anx, Dep	GAD-7, PHQ-9	March	Yes	-	Undergrad	Cross.
Nihmath Nisha et al. (149)	359	-	50.40	India	Anx, Dep	GAD-7, CES-D	April—June	Yes	Yes	Postgrad	Cross.
Nishimura et al. (150)	473	22.00	65.80	Japan	Anx, Dep	GAD-7, PHQ-9	June	Yes	Yes	Postgrad	Cross.
Nomura et al. (151)	2,449	20.50	53.80	Japan	Dep	PHQ-9	May—June	Yes	-	-	Cross.
Padron et al. (152)	932	-	23.80	Spain	Anx, Dep	GAD-7, PHQ-9	April—May	Yes	-	Undergrad	Cross.
Pandey et al. (153)	82	-	43.40	India	Anx, Dep	GAD-7, PHQ-9	April	Yes	Yes	Postgrad	Cross.
Patelarou et al. (154)	787	22.70	16.10	Greece, Spain, Albania	Dep	PHQ-9	April—May	Yes	Yes	Undergrad	Cross.
Patsali et al. (155)	1,535	22.00	28.08	Greece	Dep	CES-D	April—May	Yes	-	Undergrad	Cross.
Pavan et al. (156)	233	22.82	58.70	India	Anx	GAD-7	August	Yes	Yes	Postgrad	Cross.
Pelaccia et al. (157)	1,165	23.00	34.80	France	Anx	STAI-S	May	Yes	Yes	Postgrad	Cross.
Poon et al. (158)	374	-	-	China	Anx, Dep	GAD-7, PHQ-9	-	Yes	Yes	Postgrad	Cross.
Qanash et al. (159)	721	22.00	40.60	Saudi Arabia	Anx, Dep	PHQ-4, PH-4	April—June	Yes	-	Undergrad	Cross.
Rogowska et al. (160)	1,512	20.06	31.35	Ukraine	Anx, Dep	GAD-7, PHQ-9	May—June	Yes	-	Undergrad	Cross.
Rogowska et al. (161)	914	23.04	56.89	Poland	Anx	GAD-7	March—April	Yes	-	Undergrad	Cross.
Romeo et al. (162)	478	23.30	22.60	Italy	Anx, Dep	STAI-Y1, BDI-II	March—April	Yes	-	Undergrad	Cross.
Rosenthal et al. (163)	222	-	8.00	USA	Anx, Dep	DASS-21	June	Yes	Yes	Postgrad	Cross.
Rudenstine et al. (164)	1,821	26.17	27.10	USA	Anx, Dep	GAD-7, PHQ-9	April—May	Yes	-	Undergrad	Cross.
Saadeh et al. (165)	6,157	19.79	28.70	Jordan	Dep	CES-D	-	Yes	-	Undergrad	Cross.
Saddik et al. (166)	1,485	20.50	28.20	UAE	Anx	GAD-7	March	Yes	Mixed	-	Long.
Safa et al. (167)	425	22.00	37.65	Bangladesh	Anx, Dep	HADS	April—May	Yes	Yes	Postgrad	Cross.
Saguem et al. (168)	251	21.00	17.50	Tunisia	Anx, Dep	DASS-21	April—May	Yes	Yes	Postgrad	Cross.
Salman et al. (169)	1,134	21.70	29.50	Pakistan	Anx, Dep	GAD-7, PHQ-9	April—May	Yes	-	Undergrad	Cross.
Saraswathi et al. (170)	217	20.00	35.94	India	Anx, Dep	DASS-21	June	Yes	Yes	Postgrad	Long.
Sathe et al. (171)	433	20.00	27.94	India	Dep	PHQ-9	-	Yes	-	Undergrad	Cross.
Savitsky et al. (172)	216	26.00	12.04	Israel	Anx	GAD-7	March—April	Yes	Yes	Undergrad	Cross.
Sayeed et al. (173)	589	-	65.70	Bangladesh	Anx, Dep	DASS-21	April—May	Yes	-	Undergrad	Cross.
Shailaja et al. (174)	530	20.57	42.6	India	Anx, Dep	DASS-21	April	Yes	Yes	Postgrad	Cross.
Sogut et al. (175)	972	20.79	9.44	Turkey	Anx	BAI	March	Yes	Yes	Undergrad	Cross.
Song et al. (176)	1,128	-	44.00	China	Anx, Dep	SAS, SDS	February	Yes	Mixed	-	Cross.
Song et al. (177)	261	20.00	46.70	China	Anx, Dep	DASS-21	-	Yes	-	-	Cross.
Soria and Horgos (178)	69,054	20.00	72.80	France	Anx, Dep	STAI-Y2	April—May	Yes	-	Undergrad	Cross.
Srivastava et al. (179)	97	19.15	47.42	India	Anx	GAD-7	-	Yes	Yes	Postgrad	Cross.
Sultana et al. (180)	3,997	21.96	61.10	Bangladesh	Dep	PHQ-9	May—June	Yes	-	Undergrad	Cross.
Sun et al. (181)	1,912	20.28	30.23	China	Anx, Dep	GAD-7, PHQ-9	March—April	Yes	-	-	Cross.
Sundarasen et al. (182)	983	-	33.60	Malaysia	Anx	SAS	April—May	Yes	-	Undergrad	Cross.
Syam et al. (183)	1,044	21.12	17.40	Indonesia	Dep	KADS-6	April	Yes	-	Undergrad	Cross.
Tang et al. (184)	2,485	19.81	39.20	China	Dep	PHQ-9	February	Yes	-	Undergrad	Cross.
Tasnim et al. (185)	3,331	21.40	59.40	Bangladesh	Anx, Dep	DASS-21	April—May	Yes	-	Undergrad	Cross.
Vahedian-Azimi et al. (7)	207	27.23	69.10	Iran	Anx, Dep	DASS-21	February—March	Yes	Yes	Postgrad	Cross.
Vala et al. (186)	250	-	44.00	India	Anx, Dep	DASS-21	-	Yes	Yes	Postgrad	Cross.
Van Der Feltz-Cornelis et al. (187)	925	27.50	26.00	UK	Anx, Dep	GAD-7, PHQ-9	May—June	Yes	-	Undergrad	Cross.
Verma (188)	131	-	48.00	India	Anx, Dep	GAD-7, PHQ-9	-	Yes	-	Undergrad	Cross.
Villani et al. (189)	501	22.90	28.54	Italy	Anx, Dep	SAS, SDS	June—July	Yes	-	Undergrad	Cross.
Vitale et al. (190)	285	-	14.03	Italy	Dep	PHQ-9	March—April	Yes	Yes	-	Cross.
Volken et al. (191)	2,363	26.00	30.20	Switzerland	Dep	PHQ-9	April—October	Yes	-	Undergrad	Long.
Wan Mohd Yunus et al. (192)	1,005	-	24.50	Malaysia	Anx, Dep	DASS-21	April	Yes	-	Undergrad	Cross.
Wang et al. (193)	1,172	-	39.08	China	Anx	SAS	Febuary—March	Yes	-	Undergrad	Long.
Wang et al. (194)	44,447	21.00	45.50	China	Anx, Dep	SAS, CES-D	January—February	Yes	-	Undergrad	Cross.
Wang et al. (195)	2,014 (Anx) 1994 (Dep)	22.88	38.36	USA	Anx, Dep	GAD-7, PHQ-9	May	Yes	-	Undergrad	Cross.
Wang et al. (196)	3,092	-	33.60	China	Anx	GAD-7	February—March	Yes	-	Undergrad	Cross.
Wathelet et al. (197)	69,054	20.00	26.10	France	Anx, Dep	STAI-Y2, BDI	April—May	Yes	-	Undergrad	Cross.
Widiyanto et al. (198)	430	-	25.12	Indonesia	Anx, Dep	GAD-7, WHO-5	May	Yes	-	Undergrad	Cross.
Wong et al. (199)	340	-	-	Malaysia	Anx, Dep	DASS-21	May—September	Yes	-	Undergrad	Cross.
Wu et al. (200)	11,787	20.45	42.89	China	Anx, Dep	GAD-7, PHQ-9	February	Yes	-	Undergrad	Cross.
Xiang et al. (201)	1,396	20.68	63.10	China	Anx, Dep	SAS, SDS	February—March	Yes	-	Undergrad	Cross.
Xiao et al. (202)	933	-	29.90	China	Anx, Dep	GAD-7, PHQ-9	February	Yes	Yes	Postgrad	Cross.
Xie et al. (203)	1,026	-	36.40	China	Dep	SDS	February	Yes	Yes	Postgrad	Cross.
Xie et al. (204)	2,705	-	22.48	China	Anx, Dep	GAD-7, PHQ-9	February	Yes	Mixed	Undergrad	Cross.
Xin et al. (205)	24,378	19.90	32.30	China	Dep	PHQ-9	February	Yes	-	Undergrad	Cross.
Yadav et al. (206)	409	22.10	16.90	Nepal	Anx, Dep	GAD-7, PHQ-9	June	Yes	No	Postgrad	Cross.
Yang et al. (207)	521	-	22.50	China	Anx	SAS	April—May	Yes	-	Undergrad	Cross.
Yu et al. (208)	430	18.51	19.30	China	Anx, Dep	GAD-7, PHQ-9	October	Yes	-	Undergrad	Cross.
Yu et al. (209)	23,863	-	31.90	China	Dep	PHQ-9	February	Yes	-	Undergrad	Cross.
Yu et al. (210)	1,681	-	35.20	China	Dep	CES-D	March	Yes	-	Undergrad	Cross.
Zhang et al. (211)	66	20.70	37.88	China	Anx, Dep	DASS-21	February—March	Yes	-	Undergrad	Long.
Zhang et al. (36)	1,041	21.34	47.60	China	Anx, Dep	DASS-21	April	Yes	Yes	Postgrad	Cross.
Zhao et al. (212)	821	23.08	37.15	China, South Korea, Japan	Dep	PHQ-9	March—April	Yes	-	-	Cross.
Zhao et al. (213)	420	22.90	31.67	China	Dep	PHQ-9	March—April	Yes	-	Undergrad	Cross.
Zhou et al. (214)	4,099	-	25.00	China	Anx, Dep	GAD-7, PHQ-9	March	Yes	-	Undergrad	Cross.
Zhu et al. (215)	342	20.72	13.20	China	Anx, Dep	GAD-7, PH-9	March—April	Yes	Yes	Undergrad	Cross.

*BAI, Beck Anxiety Inventory; BDI, Beck Depression Inventory; BDI-II, Beck Depression Inventory-II; CES-D, Center for Epidemiologic Studies Depression Scale; DASS-18, Depression, Anxiety and Stress Scale 18-Item; DASS-21, Depression, Anxiety and Stress Scale 21-Item; DASS-42, Depression, Anxiety and Stress Scale 42-Item; GHQ-28, General Health Questionnaire-28; GAD-7, Generalized Anxiety Disorder 7-Item; HADS, Hospital Anxiety and Depression Scale; HAM-A, Hamilton Anxiety Rating Scale; KADS-6, Kutcher Adolescent Depression Scale 6-Item; PHQ-4, Patient Health Questionnaire 4-Item; PHQ-8, Patient Health Questionnaire 8-Item; PHQ-9, Patient Health Questionnaire 9-Item; PROMIS, Patient-Reported Outcomes Measurement Information System; PROMIS-D, PROMIS Depression Short Form; SAS, Zung Self-Rating Anxiety Scale; SDS, Zung Self-Rating Depression Scale; STAI-S, State-Trait Anxiety Inventory State Subscale; STAI-Y1, State-Trait Anxiety Inventory Form Y1; STAI-Y2, State-Trait Anxiety Inventory Form Y2; STAI-6, State-Trait Anxiety Inventory 6-Item; WHO-5, World Helath Organization Well-being Index; -, not reported*.

a *Sample size entered into the meta-analysis*.

b *Data collection for all included studies occurred in 2020*.

c *Undergrad: includes university undergraduate students, university students generally, college students generally, midwifery students, and nursing students; Postgrad: includes graduate students, medical students, dental students, pharmacy students, fellows, trainees, and postdocs*.

### Pooled Prevalence of Clinically Elevated Depressive Symptoms During COVID-19

**Figure 2:**
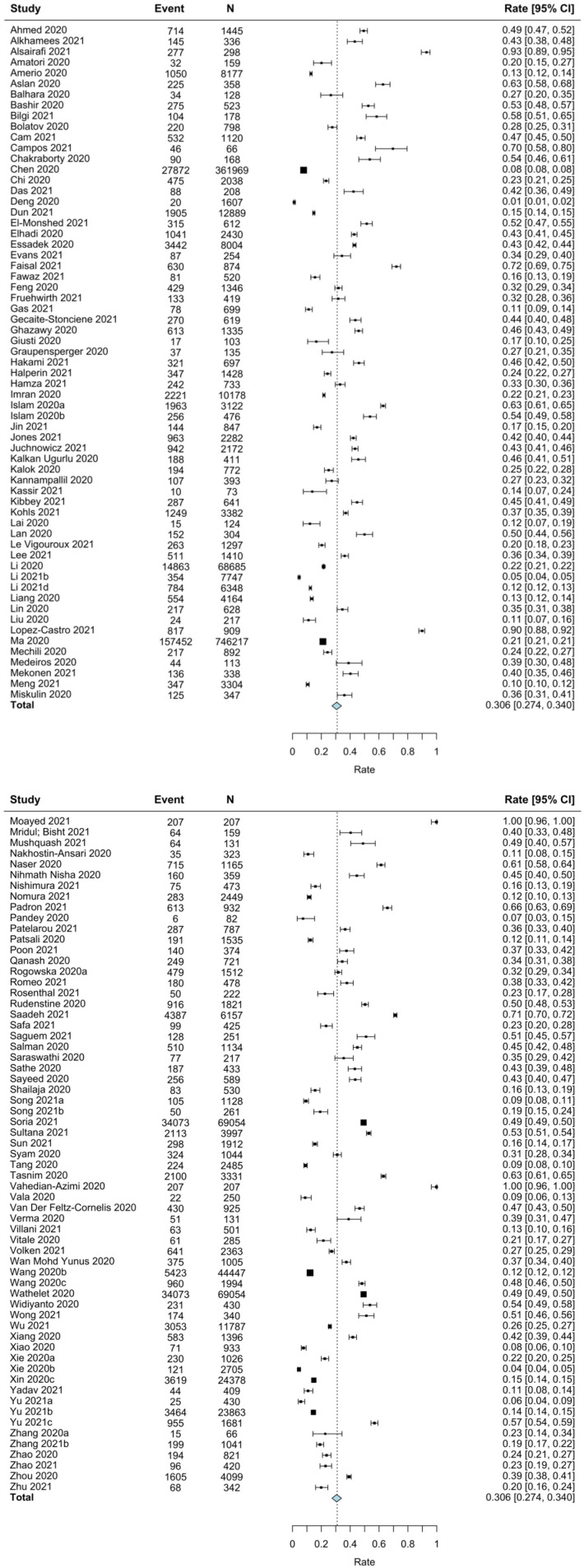
Forest plot for the meta-analysis on prevalence rates of depression in students.

A random-effects meta-analysis of 126 studies revealed a pooled event rate of 0.306 (95% CI: 0.274, 0.340; see Figure 2). That is, the prevalence of clinically significant depression across studies was 30.6%. The funnel plot was symmetrical (see Supplementary Figure 1); however, Egger's test was significant (*p* = 0.028), indicating possible publication bias. There was significant between-study heterogeneity (*Q* = 128,577.686, *p* < 0.001, *I*^2^ = 99.90); thus, potential moderators were explored based on all included studies (see Table 2).

**Table 2:** Results of moderator analyses for the prevalence of depressive symptoms in post-secondary students during COVID-19.

**Categorical moderators**	***k***	**Prevalence**	**95% CI**	**Heterogeneity *Q***	***p***
Study quality score^a^				1.948	0.163
2–3	53	0.334	0.284, 0.389		
4	73	0.287	0.247, 0.330		
Type of student				0.567	0.451
Non-healthcare	86	0.324	0.284, 0.366		
Healthcare	37	0.295	0.238, 0.360		
Student level of training				1.344	0.246
Undergraduate/College	86	0.325	0.285, 0.368		
Graduate/Professional/Fellow/Trainee	31	0.279	0.219, 0.348		
Geographical region				102.286	<0.001
North America	13	0.409^*^	0.331, 0.492		
East Asia	39	0.168^***^	0.143, 0.197		
Europe	25	0.328^***^	0.277, 0.383		
**Continuous moderators**	***k***	**Estimate**	**95% CI**	***Z***	***p***
Participant age	80	0.091	−0.003, 0.185	1.89	0.058
Participant sex	120	0.001	−0.010, 0.012	0.22	0.829
Month of data collection in 2020	119	0.157^***^	0.084, 0.230	4.20	<0.001

*k, number of studies; CI, confidence interval*.

* *p < 0.05;*

*** *p < 0.001*.

a *Four studies had a study quality of 2 and were combined with those with a study quality of 3*.

Two moderators emerged as significant: geographical region and month of data collection. Specifically, prevalence of clinically significant depression was lower in studies conducted in East Asia (*k* = 39; rate = 0.168, 95% CI: 0.143, 0.197; *p* < 0.001) compared to studies from all other regions. The second significant moderator was month of data collection, such that for every 1-month increase, a 0.16% increase in depression prevalence was observed (*k* = 119; rate = 0.157, 95% CI: 0.084, 0.230; *p* < 0.001. None of age, sex, type of student, level of training, or study quality emerged as significant moderators for the prevalence of depression symptoms among students during the COVID-19 pandemic.

### Pooled Prevalence of Clinically Elevated Anxiety Symptoms During COVID-19

**Figure 3:**
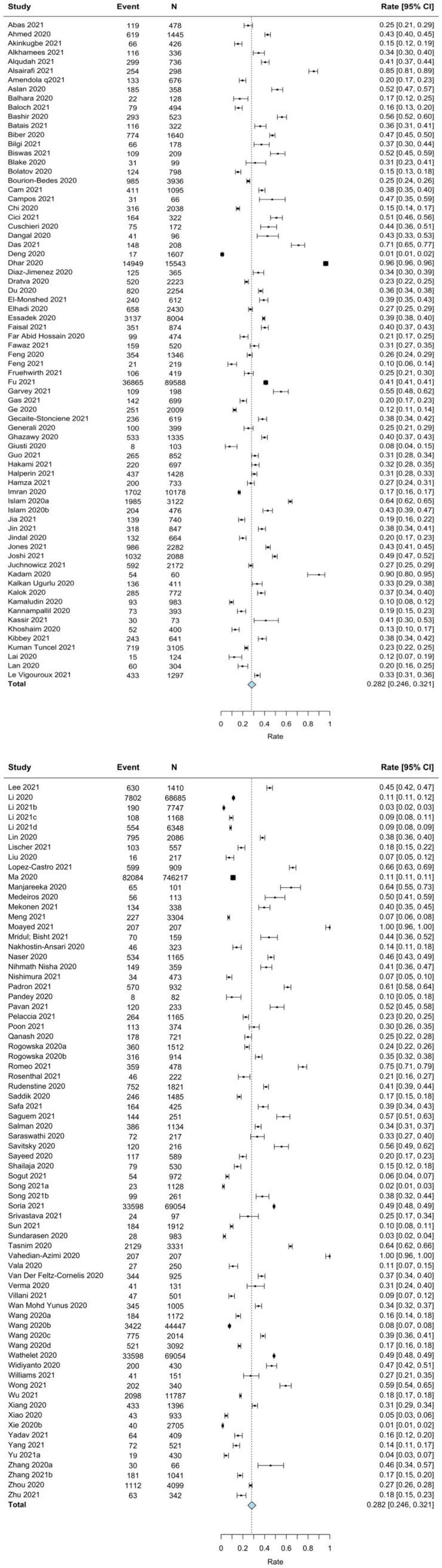
Forest plot for the meta-analysis on prevalence rates of anxiety in students during COVID-19.

A random-effects meta-analysis of 144 studies revealed a pooled event rate of 0.282 (95% CI: 0.246, 0.321; Figure 3). That is, the prevalence of clinically significant anxiety across studies was 28.2%. The funnel plot was symmetrical (see Supplementary Figure 2); however, Egger's test was significant (*p* = 0.037), indicating possible publication bias. There was significant between-study heterogeneity with (*Q* = 160,472.80, *p* < 0.001, *I*^2^ = 99.91); thus, potential moderators were explored based on all included studies (see Table 3).

**Table 3:** Results of moderator analyses for the prevalence of anxiety symptoms in post-secondary students during COVID-19.

**Categorical moderators**	***k***	**Prevalence**	**95% CI**	**Heterogeneity *Q***	***p***
Study quality score				0.237	0.627
2–3	67	0.292	0.239, 0.353		
4	77	0.273	0.225, 0.328		
Type of student				0.157	0.692
Non-healthcare	93	0.299	0.253, 0.349		
Healthcare	47	0.282	0.220, 0.353		
Student level of training				0.003	0.953
Undergraduate/College	92	0.283	0.238, 0.333		
Graduate/Professional/Fellow/Trainee	39	0.281	0.213, 0.360		
Geographical region				62.525	<0.001
East Asia	36	0.131^***^	0.101, 0.168		
North America	14	0.338^***^	0.243, 0.448		
Europe	30	0.314^***^	0.250, 0.386		
**Continuous moderators**	***k***	**Estimate**	**95% CI**	***Z***	***p***
Participant age	83	0.057	−0.026, 0.141	1.35	0.177
Participant sex	137	0.003	−0.010, 0.016	0.41	0.679
Month of data collection in 2020	133	0.178^***^	0.113, 0.243	5.34	<0.001

*k, number of studies; CI, confidence interval*.

*** *p < 0.001*.

Two moderators emerged as significant: geographical region and month of data collection. Specifically, the prevalence of clinically significant anxiety symptoms was lower among studies conducted in East Asia compared to all other geographical regions (*k* = 36; rate = 0.131, 95% CI: 0.101, 0.168; *p* < 0.001). Additionally, for every 1-month increase, a 0.18% increase in anxiety prevalence was observed (*k* = 133; rate = 0.178, 95% CI: 0.113, 0.243; *p* < 0.001). None of age, sex, type of student, level of training, or study quality emerged as significant moderators for the prevalence of clinically significant anxiety symptoms among students during the COVID-19 pandemic.

## Discussion

In the current meta-analysis, the pooled estimates of post-secondary students who reported clinically elevated depression (*N* = 126 studies) or anxiety (*N* = 144 studies) symptoms were 30.6 and 28.2%, respectively. Although findings of the present research indicate estimates are generally consistent with estimates prior to the COVID-19 pandemic, which ranged from 23.8 to 33% (9, 10, 12), anxiety and depression among post-secondary students remains a cause for significant concern. First, the rates of clinically significant anxiety and depression observed among post-secondary students during the COVID-19 pandemic were notably higher among students compared to the general population (216, 217) and continue to be higher relative to other populations during the COVID-19 pandemic [e.g., (4, 148)]. Second, in addition to the COVID-19 related stressors faced uniquely by student populations [e.g., academic disruptions and uncertainty; (19)], they also experienced many of the risk factors that have been attributed to worsened mental health among the general population, including financial insecurity, unemployment, and loss of loved ones (2). Indeed, post-secondary student populations lie at a unique intersection of elevated risk for mental health difficulties during the COVID-19 pandemic. Overall, results herein highlight the importance of continued investigation into who is struggling as well as which factors can be targeted through mental health intervention. For example, it will be important for future research to follow participants longitudinally to determine if current levels of anxiety and depression decrease, increase, and/or are sustained over time.

Although it may appear as though global estimates of mental health concerns in this population appear to have remained largely unchanged compared to pre-pandemic estimates, it is of utmost importance to consider the heterogeneous trajectories of mental health during the COVID-19 pandemic. That is, while the mental health of some students may have remained stable prior to, and during the pandemic, the pandemic may have initiated and/or attenuated mental distress in other students. Previous research has shown disparities in who was more severely impacted during the COVID-19 pandemic from a mental health standpoint (218). Recent studies showed that students who faced greater COVID-19 related stressors (e.g., lack of social support, uncertainties about academic programs) were more vulnerable to declines in mental health (122). Thus, whereas some students may have experienced consistent or improved mental health, it is likely that those with greater stressors may be disproportionately negatively impacted by the COVID-19 pandemic. It will be important in future longitudinal research to examine the trajectories of mental distress from pre-pandemic to during the pandemic (and beyond) to ascertain a more complete picture of the patterns of stability and change in mental distress among post-secondary students.

We included a much larger sample of studies (*n* = 176, ~2 million participants) and applied more strict inclusion criteria in the current study, compared to previous meta-analyses. More specifically, we only included studies that reported clinically elevated depression and anxiety symptoms (i.e., above clinical cut-offs in the moderate to severe range), whereas previous meta-analyses have also included mild (i.e., subthreshold) symptoms in their pooled prevalence estimates, which could lead to estimate inflation. Nonetheless, the current prevalence estimates are in line with previous meta-analyses examining post-secondary student depressive [26–34%; (21–24)] and anxiety [28–31%; (21, 24, 25)] symptoms during the COVID-19 pandemic. However, unique to this meta-analysis was an examination of moderator variables. Results revealed that geographical location and month of data collection were important for explaining between-study differences in prevalence estimates, with rates of both anxiety and depression being lower in East Asian countries and higher as the month of data collection increased. Further, while estimates of mental illness typically vary by sex and age, these demographic factors did not explain between-study variability in the current meta-analysis of pandemic related mental illness symptoms, emphasizing the importance of providing adequate mental health services to individuals regardless of age or sex. As well, study quality was not a significant moderator. This may be related to the fact that there was limited variability in study quality among included studies (2–4 out of 5 with a mean study quality of 3.5). Although previous studies have found differences in student mental illness depending on level of study before (219) and during the COVID-19 pandemic (29), and healthcare fields may be disproportionately affected by the pandemic, none of these emerged as significant moderators. This finding may be explained by the fact that students working in healthcare fields may not necessarily be in direct contact with COVID-19 patients. Further, there may be stressors that negatively impact all students, regardless of level of training and type of student, such as financial stress.

This meta-analysis suggests that rates of clinically significant anxiety and depression among post-secondary students may be similar to pre-pandemic estimates. It is possible that the COVID-19 pandemic may have led to a shift in university and college procedures that created favorable learning conditions for post-secondary students. Take, for example, the finding that a sample of medical students reported lower levels of burnout during online learning over the course of the pandemic compared to traditional in-person learning pre-pandemic (85). As such, factors such as method of teaching delivery could have created an environment for students that decreases stress and increases flexibility and accessibility compared to in-person learning pre-pandemic.

Rates of anxiety and depression may also have remained relatively unchanged due to continued access to familial social support. Research during the pandemic has shown that college students who reported greater social support displayed better psychological health compared to those with lower levels of social support (122, 220). Many post-secondary students moved home and were in quarantine with family members. Returning home may have provided a source of support that helped to protect against the adverse mental health consequences of the pandemic, given that students who did not return to their home country or region reported more COVID-19 related stressors, including a lack of social support and worse mental health (122). For all students, access to social media may have been a particularly helpful tool to continue seeking and obtaining social support from peers, relatives, and colleagues (221).

Further, despite the disruption to mental health services during COVID-19 generally, many post-secondary students may have been able to continue to receive mental health services. Even prior to the pandemic, some colleges began implementing telehealth services to meet the increasing demands and these telemental health services may have been particularly helpful for students by allowing them to stay connected to care (222). Previous research has shown that many students, especially those with greater levels of depression and anxiety symptoms, are willing to use telemental health resources (223). Lastly, the COVID-19 pandemic has highlighted the importance of accessible mental health services and some institutions may be presently exploring strategies to promote better mental health among their students [e.g., (224, 225)].

Many included studies with the largest sample sizes were conducted in East Asian countries. The current results revealed that samples from East Asia possessed lower pooled prevalence rates of depression and anxiety compared to other geographical regions. Previous research has documented that East Asian populations may underreport or underestimate their psychological distress (32), either because they do not perceive their symptoms as indicative of mental health problems or due to the stigma associated with mental illness. Thus, the large representation of studies from East Asian countries should be considered in the interpretation of the minimal increase in results from pre- to during the COVID-19 pandemic. Furthermore, East Asian countries were also the first to report COVID-19 infections and had some of the strongest public health measures. The measures to “flatten the curve” may have reduced the risk of mental health responses where infection rates were diminished. These results are consistent with existing literature that similarly found rates of anxiety and depression among youth were lower in East Asian countries during COVID-19 (4). The current meta-analysis cannot explicate whether regional differences in the prevalence of anxiety and depression symptoms were related to true cultural differences in these symptoms, or to differing attitudes and reports of symptoms.

In addition to geographical region, the current study revealed month of data collection as a moderator of elevated depression and anxiety, such that rates of depression and anxiety increased later into the COVID-19 pandemic. This finding parallels a recent meta-analysis on children and adolescents (4), which also found that mental health deteriorated over the course of the pandemic. Among young adults, peer relationships can be an important element of social support (20). Although students may have experienced increased familial support throughout the COVID-19 pandemic, campus closures and social distancing measures removed students from a critical source of social support (i.e., peers). One possible explanation for the current finding is that social isolation, campus closures, and academic disruptions had a compounding effect on the mental health of post-secondary students as the COVID-19 pandemic progressed (14, 19). Alternatively, studies conducted earlier in the COVID-19 pandemic were more likely to have been conducted in East Asia as East Asian countries were the first to report COVID-19 infections (Racine et al., 2021). Previous studies have indicated that self-reported prevalence of psychological distress tends to be lower among East Asian populations (226).

### Limitations

The results of this meta-analysis should be viewed within the context of several limitations. First, power was limited in some categorical moderator analyses due to small sample sizes at each level of the moderator variable. Several potentially interesting moderators could also not be explored as there were insufficient studies reporting on these factors. For example, factors that may have increased or decreased prevalence rates of anxiety and depression could include SES, history of pre-existing mental disorder, and living situation (e.g., subjected to stay-at-home vs. physical distancing orders). Indeed, pandemic-related mental health research has shown that mental illness tends to increase during periods of quarantine and self-isolation. A fuller exploration of these factors in future research will be essential for planning and targeting interventions to address mental distress. Relatedly, despite strict criteria for inclusion in the present meta-analysis (e.g., use of clinical cut off scores for depression and anxiety), there was still considerable heterogeneity among the included studies that was not accounted for by the tested moderators. This indicates there is notable heterogeneity in research conducted on this topic to date, suggesting there may be unexplored moderators that further account for the observed heterogeneity. Future research may wish to explore moderators including SES, vaccination rates, and mental health assessment measures to determine if greater heterogeneity among existing research can be accounted for. Second, while all included studies used validated measures of anxiety and depressive symptoms, no study to date has employed diagnostic measures. Therefore, our results are based on elevated self-reports of moderate to severe anxiety and depressive symptoms, but not diagnoses of these disorders. Fourth, all included studies are cross-sectional reports of mental illness symptoms. Cross-sectional studies can establish rates of mental illness during an acute period of distress, but it is critical to establish if the estimated prevalence rates are sustained over time.

### Future Directions

This meta-analysis provided a synthesis of existing evidence on clinically elevated depressive and anxiety symptoms experienced by post-secondary students during the COVID-19 pandemic. Future research should attend to several methodological issues to inform this body of research more fully and to increase the applicability of findings for health policy and practice (32, 227). First, as aptly outlined by others (2, 32), more rigorous recruitment methods, such as random sampling methods, are critical in order to fully understand the burden of the COVID-19 pandemic and capture inequalities experienced by vulnerable groups. Second, it is important for future research to continue to longitudinally examine whether the prevalence of anxiety and depressive symptoms remain constant, decrease, or increase over the course of the pandemic, and beyond. For example, an innovative study by Ayers et al. (228) demonstrated that internet searches for acute anxiety spiked early in the pandemic compared to historical pre-pandemic levels, but following the peak of the pandemic, searches returned to historical pre-pandemic levels. To date, several longitudinal studies have been conducted to assess mental illness throughout the COVID-19 pandemic [e.g., (3, 229, 230)]. For example, emerging longitudinal research on student populations by Amendola et al. (50) shows that the prevalence of moderate-to-severe anxiety symptoms during the COVID-19 pandemic decreased between the first to second timepoint. As highlighted above, the present research underscores the need for additional longitudinal research on mental illness among post-secondary student populations over the course of, and in the aftermath of, the COVID-19 pandemic to determine if estimates are sustained over time and/or lead to an increase in treatment seeking. Cohort samples with baseline estimates pre-COVID-19 pandemic are particularly advantageous, as they can ascertain changes in prevalence rates on account of the COVID-19 pandemic. Future longitudinal studies can also be harnessed to examine mechanisms associated with mental health, so that targets of interventions can be mechanistically informed (2).

Future research should explore additional contextual factors that may impact the risk for mental illness. For example, student SES may have notable impacts on the ability to engage in online learning. Consider the fact that stable internet connection, electronic devices, and a workspace at home are all prerequisites to partaking in online learning. Indeed, high SES has been found to be a protective factor following natural disasters and low SES students tended to report higher rates of anxiety during the COVID-19 pandemic (231, 232). Examination of such factors may inform how best to support students and gain a better understanding regarding how to target prevention and intervention efforts. Further, targeted research with post-secondary students who have pre-existing mental illness and may be particularly impacted by COVID-related stressors [e.g., loss of social capital, suspension of mental health services; (233)] is critical to determine if these stressors have exacerbated mental illness or increased the potential for relapse (16). Initial research has found that female university students with pre-existing mental illness reported greater loneliness, avoidant, and negative emotional coping during the pandemic compared to those without pre-existing mental illness (234). Finally, to our knowledge, few studies have examined protective factors that may mitigate the risk for mental illness during the COVID-19 pandemic. Sun et al. (181) found that, among a sample of university students, perceived social support and mindfulness was associated with lower anxiety and depression symptoms. It will be important to conduct additional research to examine whether the protective benefits of social support differ between physical and virtual social support, for example, and can buffer the effects of the COVID-19 pandemic on mental health, to further inform policy and resource planning.

### Implications for Policy and Practice

The current results implicate a need for continued, and possibly increased, availability of mental health services to meet the needs of students who develop or continue to experience pre-existing mental health symptomatology during, and following, the COVID-19 pandemic. Previous research has shown that unaddressed mental health difficulties can lead to poor long-term health (235), as well as lost income and productivity (236). Distress and anxiety related to unemployment or fear of contracting illness may be best addressed *via* broader social or public health interventions, rather than psychiatric care. Thus, governments and policymakers must prioritize the funding and provision of mental health services alongside social and public health interventions that broadly improve quality of life.

Mental health supports for post-secondary students are of utmost importance given the high rates of clinically significant anxiety and depression both prior to and during the COVID-19 pandemic. For example, it may be necessary to provide students with psychoeducational materials regarding mental health and well-being (i.e., importance of sleep hygiene, routines, exercise) and create increased accessibility to in-person and/or telemental health services. Telemental health services in particular will be important to increase equitable accessibility and improve scalability for student populations (237). Further, academic accommodations, including flexible deadlines and the option of virtual lectures, for students suffering from severe mental distress should be implemented in post-secondary institutions. The mental health needs of some students may surpass what can be provided by on-campus mental health centers, and funding for students to access mental health services in the community may be necessary. Given that stress is a primary precipitant of mental illness (238), policies that reduce stress by offering students financial support (i.e., income supplements) and social support (e.g., peer support resources; helplines) may be necessary and represent important mental health prevention efforts (239). Overall, these suggestions are encouraged both during, and following, the COVID-19 pandemic. Finally, while the implementation of quarantine may be necessary at times, previous research suggests that quarantine is associated with psychological distress (14), and as such, the closure of post-secondary institutions should be considered a last resort.

## Conclusions

The current meta-analysis of 176 studies and close to 2 million participants demonstrate consistent prevalence rates of clinically elevated depressive and anxiety symptoms prior to, and during, the COVID-19 pandemic among post-secondary students. The COVID-19 pandemic represents a global crisis, both with respect to its physical consequences, but also its dire implications for the mental health of individuals globally. As such, the results of the current study represent a clarion call for urgent and sustained funding and support for evidence-based mental health screening, case-finding, and treatment for depression and anxiety.

## Data Availability Statement

The original contributions presented in the study are included in the article/Supplementary Materials, further inquiries can be directed to the corresponding author/s.

## Author Contributions

NR, SM, and JZ: concept and design. JZ, NR, RE, KD, and SM: critical revision of the manuscript for important intellectual content. NR: statistical analysis. SM: administrative, technical, and material support. NR and SM: supervision. All authors: acquisition, analysis, interpretation of data, and drafting of the manuscript.

## Conflict of Interest

The authors declare that the research was conducted in the absence of any commercial or financial relationships that could be construed as a potential conflict of interest.

## Publisher's Note

All claims expressed in this article are solely those of the authors and do not necessarily represent those of their affiliated organizations, or those of the publisher, the editors and the reviewers. Any product that may be evaluated in this article, or claim that may be made by its manufacturer, is not guaranteed or endorsed by the publisher.
