# Supplementary material for: Post-secondary Student Mental Health During COVID-19: A Meta-Analysis
Source: Front Psychiatry. 2021 Dec 10;12:777251. doi: 10.3389/fpsyt.2021.777251 (PMC8709535; doi:10.3389/fpsyt.2021.777251)

Supplementary Table 1

Search Strategy from EMBASE (Ovid)

Embase <1974 to April 30, 2021>

1	student/ or athletic training student/ or college student/ or disabled student/ or foreign student/ or graduate student/ or exp health student/ or non-medical student/ or nontraditional student/ or phd student/ or postgraduate student/ or research student/ or social work student/ or student athlete/ or undergraduate student/ or university student/ or veterinary student/
2	exp student attitude/ or student burnout/
3	(student* or undergrad* or graduate* or learner* or freshm?n or sophomore* or junior* or pupil or pupils).tw,kw.
4	1 or 2 or 3
5	mental health/ or community mental health/ or psychological well-being/ or emotion regulation/ or psychological adjustment/
6	mental disease/ or adjustment disorder/ or alexithymia/ or exp anxiety disorder/ or emotional disorder/ or exp neurosis/ or exp psychotrauma/
7	mood disorder/ or affective neurosis/ or major affective disorder/ or minor affective disorder/
8	behavior disorder/
9	depression/ or agitated depression/ or atypical depression/ or chronic depression/ or depressive psychosis/ or dysphoria/ or dysthymia/ or endogenous depression/ or major depression/ or masked depression/ or melancholia/ or minor depression/ or "mixed anxiety and depression"/ or organic depression/ or reactive depression/ or recurrent brief depression/ or subsyndromal depression/or treatment resistant depression/
10	stress/ or acute stress/ or adaptation syndrome/ or behavioral stress/ or exp burnout/ or exp chronic stress/ or critical incident stress/ or early life stress/ or emotional stress/ or family stress/ or home stress/ or interpersonal stress/ or life stress/ or mental stress/ or role stress/ or school stress/ or exp social stress/
11	anxiety/ or anticipatory anxiety/
12	psychological resilience/ or exp coping behavior/ or wellbeing/

13	((mental* or psych* or behavior* or behaviour* or mood* or panic* or affective or emotion* or neurotic or adjustment or reactive) adj2 (health* or ill* or well* or disease* or disorder* or syndrome* or hygiene or symptom* or disturbance* or dysfunction* or attack* or trauma* or condition* or state* or status* or adapt* or regulat* or adjust* or control*)).tw,kw.
14	(anxiet* or depress* or stress* or distress* or PTSD or posttraumatic or post traumatic or nervous* or neuroses or neurosis or catastrophi* or psychoneuros?s or psycho-neuros?s or phobi* or cyclothymi* or melanchol* or trauma* or obsess* or OCD or dysthymi* or dysphori* or astheni* or psychastheni* or alexithymi* or hysteri* or psychotrauma or burnout or burn-out or cothymi* or self-harm* or suicid* or parasuicid* or self-injur* or self-mutilat* or self-destructive behavio?r* or well-being or wellbeing or resilien*).tw,kw.
15	5 or 6 or 7 or 8 or 9 or 10 or 11 or 12 or 13 or 14
16	coronavirus disease 2019/ or coronavirus infection/
17	(2019-ncov* or 2019ncov* or 2019n-cov* or coronaviru* or corona viru* or covid or covid-19 or covid19* or ncov* or n-cov* or novelcov* or covid-2019 or covid2019 or SARS-COV-2* or SARS-COV2* or SARSCOV-2* or SARSCOV2* or SARSCOV19 or SARS-COV-19 or SARSCOV-19 or SARS-COV-2019 or SARSCOV2019 or SARSCOV-2019 or Wuhan pneumonia or Wuhan virus or severe acute respiratory syndrome or severe acute respiratory disease).tw,kw.
18	16 or 17
19	4 and 15 and 18

Note. *Indicates that words were truncated it order instruct the database to capture all variant endings and spellings of the search terms.

Supplementary Table 2

Search Strategy from APA PsycInfo (Ovid)

APA PsycInfo <1806 to April Week 4 2021>

1	students/ or business students/ or exp college students/ or dental students/ or graduate students/ or international students/ or law students/ or medical students/ or postgraduate students/ or reentry students/ or seminarians/ or transfer students/ or vocational school students/
2	student characteristics/ or student attitudes/
3	student attrition/
4	(student* or undergrad* or graduate* or learner* or freshm?n or sophomore* or junior* or pupil or pupils).tw.
5	1 or 2 or 3 or 4
6	exp mental health/
7	mental disorders/ or exp affective disorders/ or exp anxiety disorders/ or exp neurosis/ or exp "stress and trauma related disorders"/
8	"depression (emotion)"/ or cyclothymic disorder/ or internalizing symptoms/
9	stress/ or chronic stress/ or environmental stress/ or physiological stress/ or post-traumatic stress/ or psychological stress/ or social stress/ or exp stress reactions/ or distress/
10	exp anxiety/
11	suicidal ideation/
12	exp self-destructive behavior/
13	emotional states/ or catastrophizing/ or emotional trauma/ or hopelessness/
14	trauma/ or post-traumatic stress/
15	emotional control/ or emotional adjustment/ or anger control/ or coping behavior/ or "stress and coping measures"/ or exp wellbeing/ or emotional regulation/ or "resilience (psychological)"/
16	((mental* or psych* or behavior* or behaviour* or mood* or panic* or affective or emotion* or neurotic or adjustment) adj2 (health* or ill* or well* or disease* or disorder* or syndrome* or hygiene or symptom* or disturbance* or dysfunction* or attack* or trauma* or condition* or state* or status* or adapt* or regulat* or adjust* or control*)).tw.

17	(anxiet* or depress* or stress* or distress* or PTSD or posttraumatic or post traumatic or nervous* or neuroses or neurosis or catastrophi* or psychoneuros?s or psycho-neuros?s or phobi* or cyclothymi* or melanchol* or trauma* or obsess* or OCD or dysthymi* or dysphori* or astheni* or psychastheni* or alexithymi* or hysteri* or psychotrauma or burnout or burn-out or cothymi* or self-harm* or suicid* or parasuicid* or self-injur* or self-mutilat* or self-destructive behavio?r* or wellbeing or well-being or resilien*).tw.
18	6 or 7 or 8 or 9 or 10 or 11 or 12 or 13 or 14 or 15 or 16 or 17
19	exp coronavirus/
20	(2019-ncov* or 2019ncov* or 2019n-cov* or coronaviru* or corona viru* or covid or covid-19 or covid19* or ncov* or n-cov* or novelcov* or covid-2019 or covid2019 or SARS-COV-2* or SARS-COV2* or SARSCOV-2* or SARSCOV2* or SARSCOV19 or SARS-COV-19 or SARSCOV-19 or SARS-COV-2019 or SARSCOV2019 or SARSCOV-2019 or Wuhan pneumonia or Wuhan virus or severe acute respiratory syndrome or severe acute respiratory disease).tw.
21	19 or 20
22	5 and 18 and 21

Note. *Indicates that words were truncated it order instruct the database to capture all variant endings and spellings of the search terms.

Supplementary Table 3

Search Strategy for Cochrane Central Register of Controlled Trials (Ovid)

EBM Reviews - Cochrane Central Register of Controlled Trials

1	exp students/
2	(student* or undergrad* or graduate* or learner* or freshm?n or sophomore* or junior* or pupil or pupils).tw,kw.
3	1 or 2
4	mental health/
5	mental disorders/ or adjustment disorders/ or exp anxiety disorders/ or exp mood disorders/ or exp depressive disorder/ or neurotic disorders/
6	affective symptoms/ or depression/ or obsessive behavior/ or exp self-injurious behavior/ or stress, psychological/
7	resilience, psychological/ or adaptation, psychological/
8	anxiety/ or catastrophization/ or panic/
9	((mental* or psychiatric or psychologic* or behavior* or behaviour* or mood* or panic* or affective or emotion* or neurotic or psychoneurotic or psycho-neurotic or adjustment or reactive) adj2 (health* or ill* or well* or disease* or disorder* or syndrome* or hygiene or symptom* or disturbance* or dysfunction* or attack* or trauma* or condition* or state* or status* or adapt* or regulat* or adjust* or control*)).tw,kw.
10	(anxiet* or depress* or stress* or distress* or PTSD or posttraumatic or post traumatic or nervous* or neuroses or neurosis or catastrophi* or psychoneuros?s or psycho-neuros?s or phobi* or cyclothymi* or melanchol* or trauma* or obsess* or OCD or dysthymi* or dysphori* or astheni* or psychastheni* or alexithymi* or hysteri* or psychotrauma or burnout or burn-out or cothymi* or self-harm* or suicid* or parasuicid* or self-injur* or self-mutilat* or self-destructive behavio?r* or well-being or wellbeing or resilien*).tw,kw.
11	4 or 5 or 6 or 7 or 8 or 9 or 10
12	exp Coronaviridae Infections/
13	exp coronaviridae/
14	(2019-ncov* or 2019ncov* or 2019n-cov* or coronaviru* or corona viru* or covid or covid-19 or covid19* or ncov* or n-cov* or novelcov* or covid-2019 or

	covid2019 or SARS-COV-2* or SARS-COV2* or SARSCOV-2* or SARSCOV2* or SARSCOV19 or SARS-COV-19 or SARSCOV-19 or SARS-COV-2019 or SARSCOV2019 or SARSCOV-2019 or Wuhan pneumonia or Wuhan virus or severe acute respiratory syndrome or severe acute respiratory disease).tw,kw.
15	12 or 13 or 14
16	3 and 11 and 15

Note. *Indicates that words were truncated it order instruct the database to capture all variant endings and spellings of the search terms.

Supplementary Table 4

Search Strategy for ERIC (EBSCOhost)

S1	TI (student* or undergrad* or graduate* or learner* or freshm?n or sophomore* or junior* or pupil or pupils) OR AB (student* or undergrad* or graduate* or learner* or freshm?n or sophomore* or junior* or pupil or pupils)
S2	TI (((mental* or psych* or behavior* or behaviour* or mood* or panic* or affective or emotion* or neurotic or adjustment or reactive) N2 (health* or ill* or well* or disease* or disorder* or syndrome* or hygiene or symptom* or disturbance* or dysfunction* or attack* or trauma* or condition* or state* or status* or adapt* or regulat* or adjust* or control*))) OR AB (((mental* or psych* or behavior* or behaviour* or mood* or panic* or affective or emotion* or neurotic or adjustment or reactive) N2 (health* or ill* or well* or disease* or disorder* or syndrome* or hygiene or symptom* or disturbance* or dysfunction* or attack* or trauma* or condition* or state* or status* or adapt* or regulat* or adjust* or control*)))
S3	TI ((anxiet* or depress* or stress* or distress* or PTSD or posttraumatic or "post traumatic" or nervous* or neuroses or neurosis or catastrophi* or psychoneuros?s or "psycho-neuros?s" or phobi* or cyclothymi* or melanchol* or trauma* or obsess* or OCD or dysthymi* or dysphori* or astheni* or psychastheni* or alexithymi* or hysteri* or psychotrauma or burnout or "burn-out" or cothymi* or "self-harm*" or suicid* or parasuicid* or "self-injur*" or "self-mutilat*" or "self-destructive behavio?r*" or "well-being" or wellbeing or resilien*)) OR AB ((anxiet* or depress* or stress* or distress* or PTSD or posttraumatic or "post traumatic" or nervous* or neuroses or neurosis or catastrophi* or psychoneuros?s or "psycho-neuros?s" or phobi* or cyclothymi* or melanchol* or trauma* or obsess* or OCD or dysthymi* or dysphori* or astheni* or psychastheni* or alexithymi* or hysteri* or psychotrauma or burnout or "burn-out" or cothymi* or "self-harm*" or suicid* or parasuicid* or "self-injur*" or "self-mutilat*" or "self-destructive behavio?r*" or "well-being" or wellbeing or resilien*))
S4	TI (("2019-ncov*" or 2019ncov* or "2019n-cov*" or coronaviru* or "corona viru*" or covid or "covid-19" or covid19* or ncov* or "novel cov*" or "covid-2019" or covid2019 or "SARS-COV-2*" or "SARS-COV2*" or "SARSCOV-2*" or SARSCOV2* or SARSCOV19 or "SARSCOV-19" or "SARSCOV-19" or "SARS-COV-2019" or SARSCOV2019 or "SARSCOV-2019" or "Wuhan

	pneumonia" or "Wuhan virus" or "severe acute respiratory syndrome" or "severe acute respiratory disease")) OR AB (("2019-ncov*" or 2019ncov* or "2019n-cov*" or coronaviru* or "corona viru*" or covid or "covid-19" or covid19* or ncov* or "novel cov*" or "covid-2019" or covid2019 or "SARS-COV-2*" or "SARS-COV2*" or "SARSCOV-2*" or SARSCOV2* or SARSCOV19 or "SARS-COV-19" or "SARSCOV-19" or "SARS-COV-2019" or SARSCOV2019 or "SARSCOV-2019" or "Wuhan pneumonia" or "Wuhan virus" or "severe acute respiratory syndrome" or "severe acute respiratory disease"))
S5	S2 OR S3
S6	S1 AND S4 AND S5

Note. *Indicates that words were truncated it order instruct the database to capture all variant endings and spellings of the search terms.

Supplementary Table 5

Search Strategy for Education Research Complete (EBSCOhost) search strategy.

S1	TI (student* or undergrad* or graduate* or learner* or freshm?n or sophomore* or junior* or pupil or pupils) OR AB (student* or undergrad* or graduate* or learner* or freshm?n or sophomore* or junior* or pupil or pupils)
S2	TI (((mental* or psych* or behavior* or behaviour* or mood* or panic* or affective or emotion* or neurotic or adjustment or reactive) N2 (health* or ill* or well* or disease* or disorder* or syndrome* or hygiene or symptom* or disturbance* or dysfunction* or attack* or trauma* or condition* or state* or status* or adapt* or regulat* or adjust* or control*))) OR AB (((mental* or psych* or behavior* or behaviour* or mood* or panic* or affective or emotion* or neurotic or adjustment or reactive) N2 (health* or ill* or well* or disease* or disorder* or syndrome* or hygiene or symptom* or disturbance* or dysfunction* or attack* or trauma* or condition* or state* or status* or adapt* or regulat* or adjust* or control*)))
S3	TI ((anxiet* or depress* or stress* or distress* or PTSD or posttraumatic or "post traumatic" or nervous* or neuroses or neurosis or catastrophi* or psychoneuros?s or "psycho-neuros?s" or phobi* or cyclothymi* or melanchol* or trauma* or obsess* or OCD or dysthymi* or dysphori* or astheni* or psychastheni* or alexithymi* or hysteri* or psychotrauma or burnout or "burn-out" or cothymi* or "self-harm*" or suicid* or parasuicid* or "self-injur*" or "self-mutilat*" or "self-destructive behavio?r*" or "well-being" or wellbeing or resilien*)) OR AB ((anxiet* or depress* or stress* or distress* or PTSD or posttraumatic or "post traumatic" or nervous* or neuroses or neurosis or catastrophi* or psychoneuros?s or "psycho-neuros?s" or phobi* or cyclothymi* or melanchol* or trauma* or obsess* or OCD or dysthymi* or dysphori* or astheni* or psychastheni* or alexithymi* or hysteri* or psychotrauma or burnout or "burn-out" or cothymi* or "self-harm*" or suicid* or parasuicid* or "self-injur*" or "self-mutilat*" or "self-destructive behavio?r*" or "well-being" or wellbeing or resilien*))
S4	TI (("2019-ncov*" or 2019ncov* or "2019n-cov*" or coronaviru* or "corona viru*" or covid or "covid-19" or covid19* or ncov* or "novel cov*" or "covid-2019" or covid2019 or "SARS-COV-2*" or "SARS-COV2*" or "SARSCOV-2*" or SARSCOV2* or SARSCOV19 or "SARSCOV-19" or "SARSCOV-19" or "SARS-COV-2019" or SARSCOV2019 or "SARSCOV-2019" or "Wuhan

	pneumonia" or "Wuhan virus" or "severe acute respiratory syndrome" or "severe acute respiratory disease")) OR AB (("2019-ncov*" or 2019ncov* or "2019n-cov*" or coronaviru* or "corona viru*" or covid or "covid-19" or covid19* or ncov* or "novel cov*" or "covid-2019" or covid2019 or "SARS-COV-2*" or "SARS-COV2*" or "SARSCOV-2*" or SARSCOV2* or SARSCOV19 or "SARS-COV-19" or "SARSCOV-19" or "SARS-COV-2019" or SARSCOV2019 or "SARSCOV-2019" or "Wuhan pneumonia" or "Wuhan virus" or "severe acute respiratory syndrome" or "severe acute respiratory disease"))
S5	S2 OR S3
S6	S1 AND S4 AND S5

Note. *Indicates that words were truncated it order instruct the database to capture all variant endings and spellings of the search terms.

Supplementary Table 6

Study Quality Evaluation Criteria

**Quality 1: Valid Measure**	Are the depression/anxiety measures validated questionnaires?<br>0-no<br>1-yes
**Quality 2: Peer reviewed**	Was the study peer reviewed?<br>0-no<br>1-yes
**Quality 3: 50% participation**	Did at least 50% of the eligible population participate?<br>0-no<br>1-yes
**Quality 4: Objective outcome**	Were the measures of mental health objective (e.g., a diagnosis) as opposed to self-report<br>0-self-report<br>1-objective
**Quality 5: Exposure time**	Did enough time elapse since COVID for there to be an impact on mental health? (2 weeks from initial onset of COVID in the country of origin)<br>0-no<br>1-yes

Supplementary Table 7

Quality Assessment of Studies Included

First Author, Year	Valid Measure	Peer Reviewed	50% Participation	Objective Outcome	Exposure Time	Total Score (0-5)
Abas 2021	1	1	0	0	1	3
Ahmed 2020	1	1	1	0	1	4
Akinkugbe 2021	1	1	1	0	1	4
Alkhamees 2021	1	1	0	0	1	3
Alqudah 2021	1	1	1	0	1	4
Alsairafi 2021	1	1	0	0	1	3
Amatori 2020	1	1	1	0	1	4
Amendola 2021	1	1	0	0	1	3
Amerio 2020	1	1	0	0	1	3
Aslan 2020	1	1	1	0	1	4
Balhara 2020	1	1	0	0	0	2
Baloch 2021	1	1	1	0	1	4
Bashir 2020	1	1	1	0	1	4
Batais 2021	1	1	1	0	1	4
Biber 2020	1	1	1	0	0	3
Bilgi 2021	1	1	0	0	1	3
Biswas 2021	1	1	1	0	1	4
Blake 2020	1	1	0	0	1	3
Bolatov 2020	1	1	0	0	1	3
Bourion-Bedes 2020	1	1	0	0	1	3
Williams 2021	1	1	0	0	1	3
Cam 2021	1	1	1	0	1	4
Campos 2021	1	1	0	0	1	3
Chakraborty 2020	1	1	1	0	1	4
Chen 2020	1	1	1	0	1	4
Chi 2020	1	1	1	0	1	4

Cici 2021	1	1	1	0	1	4
Cuschieri 2020	1	1	1	0	1	4
Dangal 2020	1	1	1	0	1	4
Das 2021	1	1	0	0	1	3
Deng 2020	1	1	1	0	1	4
Dhar 2020	1	1	0	0	1	3
Diaz-Jimenez 2020	1	1	0	0	1	3
Dratva 2020	1	1	0	0	1	3
Du 2020	1	1	0	0	1	3
Dun 2021	1	1	1	0	1	4
Elhadi 2020	1	1	1	0	1	4
El-Monshed 2021	1	1	0	0	1	3
Essadek 2020	1	1	0	0	1	3
Evans 2021	1	1	0	0	1	3
Faisal 2021	1	1	1	0	1	4
Far Abid Hossain 2020	1	1	1	0	1	4
Fawaz 2021	1	1	1	0	1	4
Feng 2020	1	1	1	0	1	4
Feng 2021	1	1	0	0	1	3
Fruehwirth 2021	1	1	1	0	1	4
Fu 2021	1	1	0	0	1	3
Garvey 2021	1	1	1	0	1	4
Gas 2021	1	1	1	0	1	4
Ge 2020	1	1	1	0	1	4
Gecaite-Stonciene 2021	1	1	0	0	1	3
Generali 2020	1	1	1	0	1	4

Ghazawy 2020	1	1	1	0	1	4
Giusti 2020	1	1	1	0	1	4
Graupensperger 2020	1	1	1	0	1	4
Guo 2021	1	1	0	0	1	3
Hakami 2021	1	1	0	0	1	3
Halperin 2021	1	1	0	0	1	3
Hamza 2021	1	1	1	0	1	4
Imran 2020	1	1	1	0	1	4
Islam 2020	1	1	1	0	1	4
Islam 2020	1	1	1	0	1	4
Jia 2021	1	1	0	0	1	3
Jin 2021	1	1	0	0	1	3
Jin 2021	1	1	0	0	1	3
Jindal 2020	1	1	0	0	1	3
Jones 2021	1	1	1	0	1	4
Joshi 2021	1	1	0	0	0	2
Juchnowicz 2021	1	1	1	0	1	4
Kadam 2020	1	1	0	0	0	2
Kalkan Ugurlu 2020	1	1	1	0	1	4
Kalok 2020	1	1	1	0	1	4
Kamaludin 2020	1	1	1	0	1	4
Kannampallil 2020	1	1	0	0	1	3
Kaparounaki 2020	1	1	0	0	1	3
Kassir 2021	1	1	0	0	1	3
Khoshaim 2020	1	1	1	0	1	4
Kibbey 2021	1	1	1	0	1	4
Kohls 2021	1	1	0	0	1	3

Kuman Tuncel 2021	1	1	1	0	1	4
Lai 2020	1	1	1	0	1	4
Lan 2020	1	1	0	0	1	3
Le Vigouroux 2021	1	1	1	0	1	4
Lee 2021	1	1	0	0	1	3
Li 2020	1	1	0	0	1	3
Li 2021b	1	1	0	0	1	3
Li 2021c	1	1	1	0	1	4
Li 2021d	1	1	1	0	1	4
Liang 2020	1	1	0	0	1	3
Lin 2020	1	1	0	0	1	3
Lischer 2021	1	1	0	0	1	3
Liu 2020	1	1	0	0	1	3
Lopez-Castro 2021	1	1	0	0	1	3
Ma 2020	1	1	1	0	0	3
Majumdar 2020	1	1	0	0	1	3
Manjareeka 2020	1	1	1	0	1	4
Mechili 2020	1	1	1	0	1	4
Medeiros 2020	1	1	1	0	1	4
Mekonen 2021	1	1	1	0	1	4
Meng 2021	1	1	0	0	1	3
Miskulin 2020	1	1	1	0	1	4
Moayed 2021	1	1	1	0	1	4
Mridul; Bisht 2021	1	1	0	0	1	3
Mushquash 2021	1	1	0	0	1	3
Nakhostin-Ansari 2020	1	1	1	0	1	4
Naser 2020	1	1	1	0	1	4

Nihmath Nisha 2020	1	1	1	0	1	4
Nishimura 2021	1	1	1	0	1	4
Nomura 2021	1	1	1	0	1	4
Padron 2021	1	1	0	0	1	3
Pandey 2020	1	1	0	0	1	3
Patelarou 2021	1	1	1	0	1	4
Patsali 2020	1	1	1	0	1	4
Pavan 2021	1	1	1	0	1	4
Pelaccia 2021	1	1	1	0	1	4
Poon 2021	1	1	0	0	0	2
Qanash 2020	1	1	1	0	1	4
Rogowska 2020a	1	1	1	0	1	4
Rogowska 2020b	1	1	1	0	1	4
Romeo 2021	1	1	1	0	1	4
Rosenthal 2021	1	1	0	0	1	3
Rudenstine 2020	1	1	0	0	1	3
Rudenstine 2021	1	1	1	0	1	4
Saadeh 2021	1	1	1	0	0	3
Saddik 2020	1	1	1	0	1	4
Safa 2021	1	1	0	0	1	3
Saguem 2021	1	1	0	0	1	3
Salman 2020	1	1	0	0	1	3
Saraswathi 2020	1	1	1	0	1	4
Sathe 2020	1	1	0	0	0	2
Savitsky 2020	1	1	1	0	1	4
Sayeed 2020	1	1	1	0	1	4
Shailaja 2020	1	1	1	0	1	4
Sogut 2021	1	1	0	0	0	2

Song 2021a	1	1	1	0	1	4
Song 2021b	1	1	0	0	0	2
Soria 2021	1	1	0	0	1	3
Srivastava 2021	1	1	0	0	1	3
Sultana 2021	1	1	0	0	1	3
Sun 2021	1	1	0	0	1	3
Sundarasen 2020	1	1	1	0	1	4
Syam 2020	1	1	1	0	1	4
Tang 2020	1	1	1	0	1	4
Tasnim 2020	1	1	1	0	1	4
Vahedian-Azimi 2020	1	1	1	0	1	4
Vala 2020	1	1	1	0	1	4
Van Der Feltz-Cornelis 2020	1	1	0	0	1	3
Verma 2020	1	1	1	0	1	4
Villani 2021	1	1	1	0	1	4
Vitale 2020	1	1	0	0	1	3
Volken 2021	1	1	1	0	1	4
Wan Mohd Yunus 2020	1	1	1	0	1	4
Wang 2020a	1	1	0	0	1	3
Wang 2020b	1	1	1	0	1	4
Wang 2020c	1	1	1	0	1	4
Wang 2020	1	1	1	0	1	4
Wathelet 2020	1	1	0	0	1	3
Widiyanto 2020	1	1	0	0	1	3
Wong 2021	1	1	0	0	1	3
Wu 2021	1	1	1	0	1	4

Xiang 2020	1	1	1	0	1	4
Xiao 2020	1	1	1	0	0	3
Xie 2020a	1	1	1	0	1	4
Xie 2020b	1	1	0	0	1	3
Xin 2020c	1	1	1	0	1	4
Yadav 2021	1	1	0	0	1	3
Yang 2021	1	1	0	0	1	3
Yu 2021a	1	1	1	0	1	4
Yu 2021b	1	1	1	0	1	4
Yu 2021c	1	1	1	0	1	4
Zhang 2020a	1	1	1	0	1	4
Zhang 2021b	1	1	1	0	1	4
Zhao 2020	1	1	1	0	1	4
Zhao 2021	1	1	1	0	1	4
Zhou 2020	1	1	0	0	1	3
Zhu 2021	1	1	0	0	1	3

Supplementary Figure 1

Funnel plot for studies included reporting clinically significant depressive symptoms

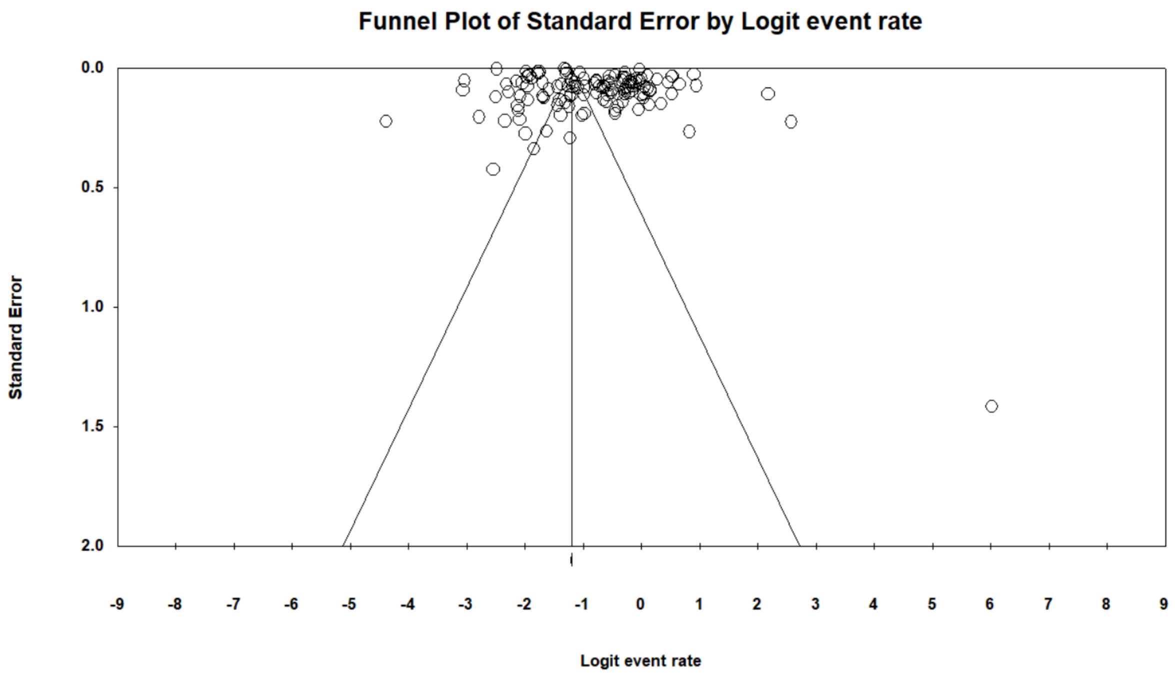

Supplementary Figure 2

Funnel plot for studies included reporting clinically significant anxiety symptoms

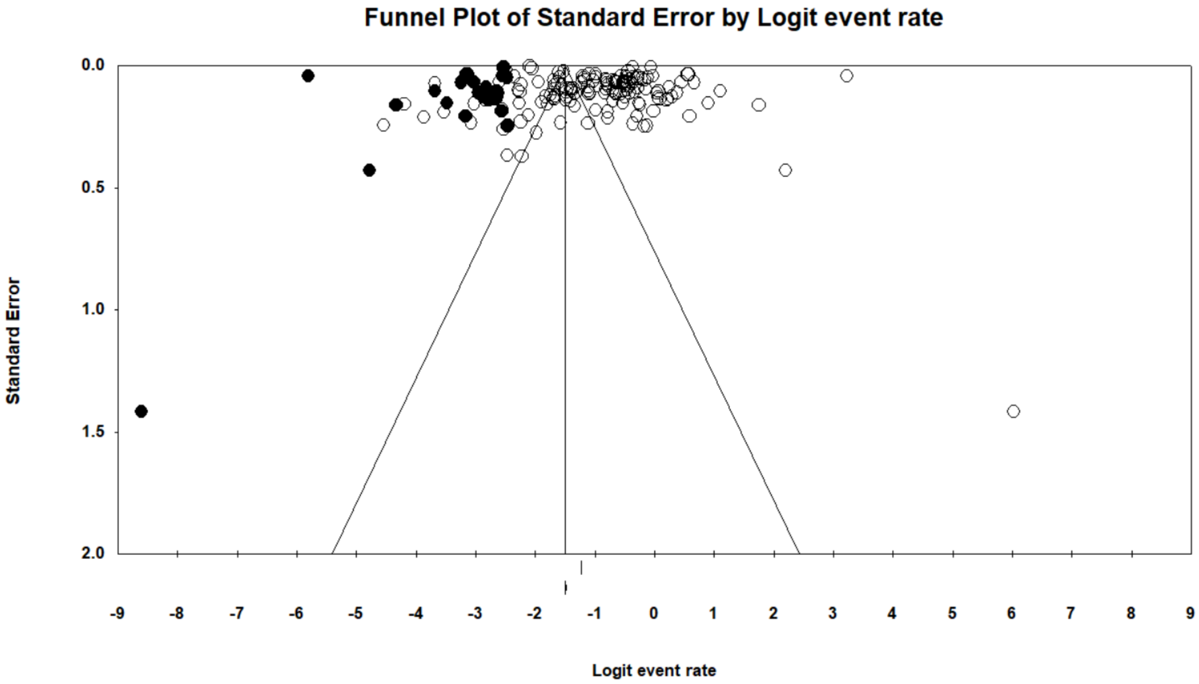

Supplement: Supplementary file 1 [file Data_Sheet_1.PDF]
